# Location and habitat specificity structure benthic microbial assemblages in a temperate seagrass ecosystem

**DOI:** 10.1128/msphere.00431-26

**Published:** 2026-08-12

**Authors:** Gabriel A. Serrano, Alejandro De Santiago, Tiago J. Pereira, Mirayana Marcelino Barros, Holly M. Bik

**Affiliations:** 1 Department of Marine Sciences, University of Georgia 1355, Athens, Georgia, USA; 2 Institute of Bioinformatics, University of Georgia 1355, Athens, Georgia, USA; Third Institute of Oceanography, Xiamen, China

**Keywords:** seagrasses, meiofauna, bacteria/archaea, 18S rRNA, 16S rRNA, metabarcoding

## Abstract

Seagrass meadows are globally distributed coastal ecosystems that provide habitats for diverse species and facilitate ecosystem services, such as carbon storage and nutrient cycling. However, anthropogenic activities and climate change are causing seagrass decline worldwide, broadly impacting ecosystem functioning. Although macrofaunal communities in seagrass habitats are relatively well studied, benthic microbial assemblages (including microeukaryote groups such as protists and microbial metazoa) remain underexplored. In this study, we used environmental DNA (eDNA) metabarcoding to characterize assemblages of bacteria/archaea (16S rRNA gene), microeukaryotes (18S rRNA amplified from raw sediment), and meiofauna (18S rRNA amplified from sieved and Ludoxed sediment fractions) in seagrass fields and bare sediment habitats at three sites along an estuarine gradient in Bodega Harbor, California. We found that microbial and microeukaryote communities were primarily structured by location, with sediment properties (sand vs silt/clay proportions) being the main driver of community assemblages. Habitat type was a secondary factor influencing benthic fauna, with bare sediments typically displaying higher biodiversity than seagrass beds, except at Westside Park mudflats, where bare sediments showed significantly reduced diversity compared to all other sampling locations. The differential recovery of nematode bioindicator taxa, including *Terschellingia*, *Daptonema*, *Viscosia*, *Microlaimus*, and *Spilophorella*, suggested that abiotic factors, such as physical disturbance, pollution, or oxygen dynamics, may influence the success of specific taxa at each site. Finally, nematode-bacterial co-occurrence networks confirmed known symbiotic associations and suggest additional taxonomic relationships to explore in future studies. Our findings underscore the importance of environmental gradients and microhabitat features in shaping benthic biodiversity in temperate seagrass ecosystems.

**IMPORTANCE:**

Seagrass meadows, which sustain a wide variety of plant and animal life, are vital coastal habitats. These submerged ecosystems support biodiversity, coastal protection, biogeochemical cycling, and carbon storage. However, seagrass habitats worldwide are declining due to pressures from climate change and human activity. This study characterizes the microbial and microeukaryote communities (including microbial animals with a body size <1 mm) that inhabit and surround temperate seagrass meadows in Bodega Harbor, California. Our findings demonstrate that location is a primary factor that determines microbial communities inhabiting sediments at each site, with habitat type (bare sediment vs seagrass beds) exerting a secondary influence on the biodiversity and distribution of microbial species. By using nematode bioindicator taxa and nematode-bacterial co-occurrence patterns, our results also suggest specific environmental factors that shape sediment biodiversity, including tidal cycles, oxygen, sulfur, and decaying organic matter. A comprehensive understanding of the variation within benthic communities associated with seagrass habitats can significantly enhance conservation and restoration efforts for seagrass meadows.

## INTRODUCTION

Seagrass habitats are distributed worldwide in shallow coastal waters and span tropical, temperate, and boreal regions, globally covering a total area of approximately 277,000 km^2^ (1–3). These highly productive ecosystems support a diverse array of species, from microorganisms to large animals, and are therefore recognized as biodiversity hotspots. Seagrasses offer shelter and food (i.e., nursery grounds) for many economically significant species of fish, crustaceans, and mollusks. They also deliver key ecosystem services, including carbon storage, sediment stabilization and accretion, and coastal protection (1, 2, 4). However, despite their high societal value, seagrasses are declining globally due to various stressors linked to anthropogenic activities and climate change, especially in the intertidal zones of low-inflow estuaries in temperate regions (4–6).

A variety of abiotic and biotic factors influence biodiversity and community structure in intertidal estuaries, including the presence of seagrasses. Studies have found that fish and benthic invertebrate communities differ between seagrass and bare sediments in both temperate and tropical regions, including changes in species abundance and biomass (7–9). Grazing and bioturbation by larger organisms can also impact seagrass density and spatial distribution, which may, in turn, affect assemblages of smaller-sized benthic species (10, 11). Other abiotic factors, such as those related to daily tide cycles and seasonality (e.g., temperature, salinity, water depth, sunlight exposure), contribute to species richness and spatial patterns of biodiversity by creating environmental gradients (12, 13). At times, these factors result in more pronounced differences among sites along the estuary than between microhabitats (i.e., seagrass vs bare sediment), highlighting the importance of spatial scale variation (12). In other cases, the differences among sites within an estuary can be just as significant as those observed between microhabitats (14, 15). In other words, abiotic factors can promote larger differences between estuarine sites versus habitat types themselves.

Most research on seagrass habitats has focused on the macrophytes themselves, including factors that control seagrass distribution and abundance, or their associations with macrofauna and megafauna such as fishes, crustaceans, and polychaetes (16). Studies of intertidal seagrass habitats have generally shown that seagrass meadows support higher macroinvertebrate diversity, richness, and density than unvegetated areas (16–18). However, Barnes (13) reported little to no effect of seagrass on benthic invertebrates near the mouth of the Kynsa estuary in South Africa, although differences between seagrass and bare sediment communities were shown to increase upstream as a result of local environmental gradients (13, 19). In terms of microscopic fauna, seagrass meadows often exhibit high meiofauna abundance and diversity, but diversity levels and species distributions are often correlated with dissolved oxygen and chlorophyll a (20, 21). Intertidal seagrass habitats have also been shown to increase the stability and richness of microbial communities (22). By creating microhabitats on plant surfaces and within seagrass patches (23, 24), generalist and specialist microbes can inhabit distinct physical niches and play key roles in biogeochemical cycling (25–28). Additionally, both emergent and submerged seagrass beds are known to vertically structure microbial and macroinvertebrate communities (27, 29), suggesting that the three-dimensional nature of benthic seagrass habitats may be a key aspect that contributes to high microbial biodiversity.

Understanding the ecological interactions between microorganisms and seagrasses is crucial for effective conservation measures (8, 16, 30, 31). However, a significant gap remains regarding benthic microbes in intertidal seagrass meadows, resulting in a poor understanding of prokaryotic and microeukaryotic biodiversity, microhabitat structure, and species co-occurrence in these habitats. Few studies have examined prokaryote and eukaryote co-occurrence in aquatic environments (27, 32–34), and even fewer have addressed these interactions specifically in seagrass habitats (35). While some correlations between prokaryotes and eukaryotes have been observed, the underlying drivers remain unclear. Recent advances in environmental DNA (eDNA) metabarcoding offer a promising approach to filling these knowledge gaps in seagrass ecosystems, particularly in mapping the spatial distribution and interactions of bacterial/archaeal and microeukaryote communities. Studies using multiplexed eDNA metabarcoding—such as nuclear 16S ribosomal RNA (rRNA) genes for bacteria and archaea, nuclear 18S rRNA genes or mitochondrial cytochrome oxidase subunit I (COI) for eukaryotes, and nuclear ITS rRNA gene regions for fungi—have begun to explore the scope of seagrass biodiversity and spatial habitat patterns, facilitating deeper insights into the distribution and relationships among prokaryotic and eukaryotic assemblages (36, 37).

In this study, we used 16S rRNA and 18S rRNA eDNA metabarcoding to characterize bacterial/archaeal and microeukaryote communities associated with seagrass and adjacent bare sediments in Bodega Harbor, northern California, USA. Bodega Harbor is a shallow, natural harbor on the Pacific Ocean, located on the eastern side of Bodega Head and formed from a geological depression within the San Andreas Fault. Bodega Harbor is protected from the larger marine water mass of Bodega Bay by a narrow sandy spit of land, with a small water channel on the western side at Campbell Cove. Ecologically, Bodega Harbor is characterized by expansive mudflats and seagrass habitats, and local benthic habitats are dominated by eelgrass (*Zostera marina*) and sea lettuce (*Ulva lactuca*). Through replicated sampling across known environmental gradients in this temperate seagrass habitat, we assessed whether community patterns for microbes and microeukaryotes reflected differences among sites across the estuary or specific microhabitats within sites. Here, we provide a significant focus on benthic meiofaunal species, a phylogenetically diverse group of marine invertebrates with a body size of <1 mm (e.g., nematodes, tardigrades, copepods, kinorhynchs, and some protist groups such as foraminifera [38]). Most meiofaunal groups are understudied due to high biodiversity and difficult taxonomy, and their spatial patterns and ecological roles are still poorly known compared to other faunal groups in seagrass habitats. We also evaluated the relationships between microbial and microeukaryote communities, focusing on nematode bioindicators and co-occurrences of bacteria and nematodes to identify potential markers of habitat types and generate hypotheses about potentially novel symbioses.

## RESULTS

### Environmental characterization of Bodega Harbor

**Fig 1:**
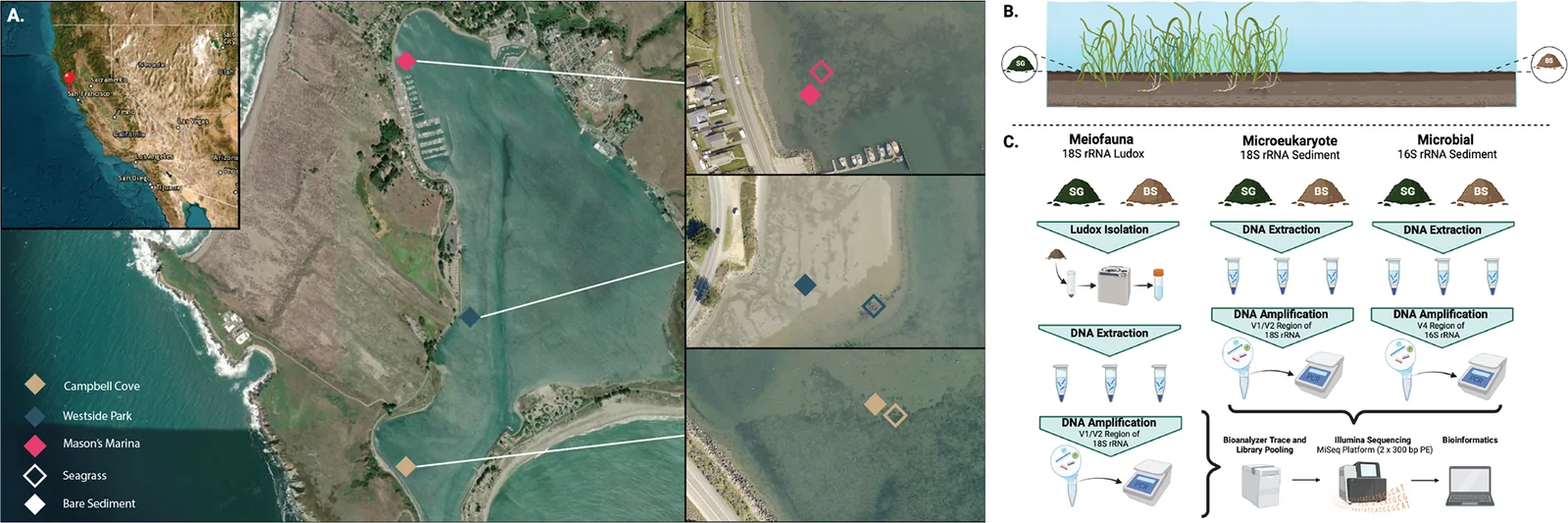
Map and schematic depicting the sampling locations and study design. (**A**) Map depicting sampling sites and microhabitats in Bodega Harbor, California, USA. Pin colors indicate Campbell Cove (tan), Westside Park (blue), and Mason’s Marina (pink). Open symbols represent seagrass, while closed symbols represent bare sediment. (**B**) Schematic depiction of seagrass vs bare sediment samples, and (C) workflow for the meiofauna, microeukaryotes, and microbial communities. Satellite imagery was obtained from the ArcGIS Map Server via ArcGIS online. Pins for Campbell Cove and Mason’s Marina were offset for clarity due to their close proximity. GPS coordinates for all sampling sites are provided in Table S1.

**Fig 2:**
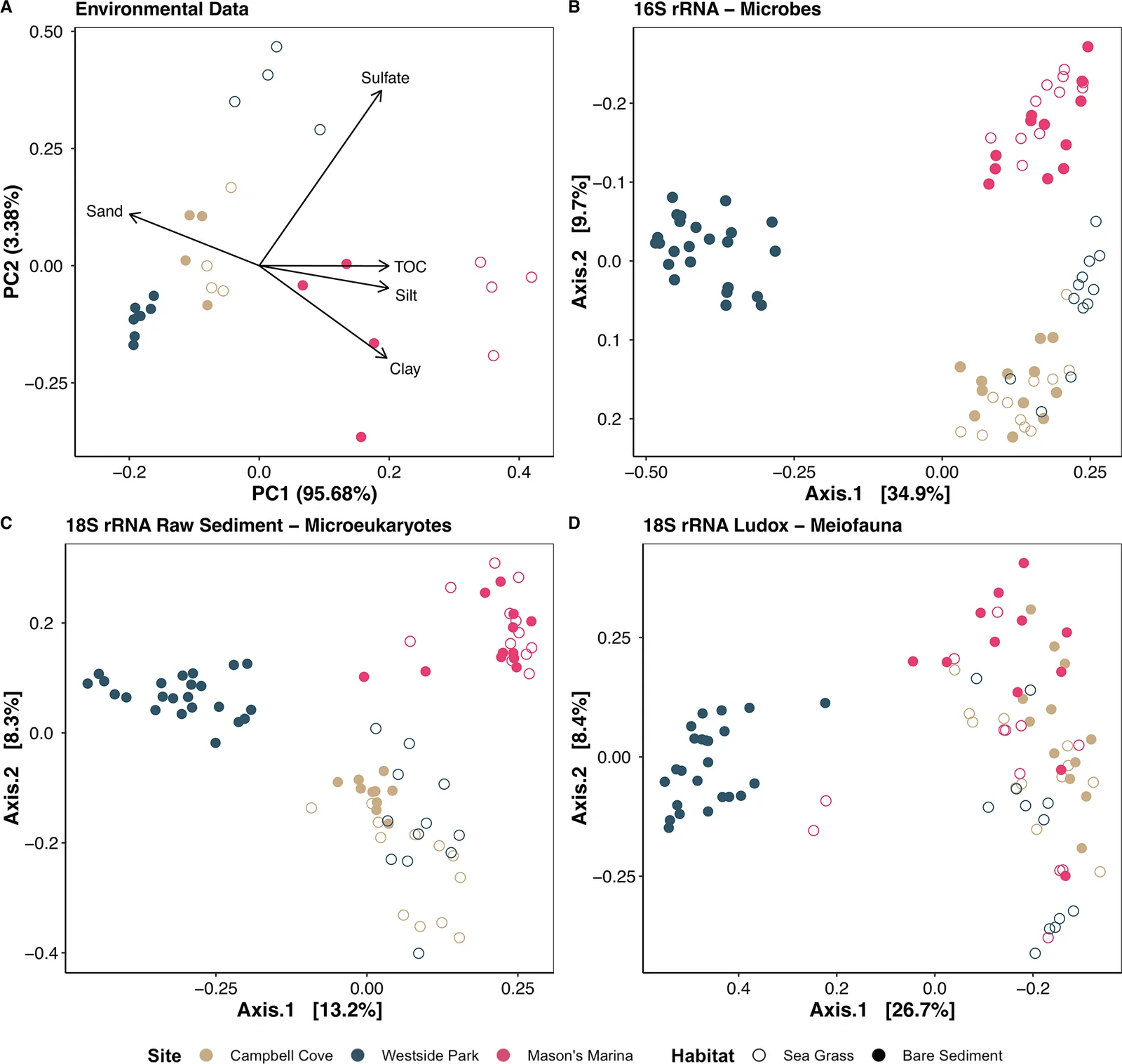
Spatial patterns across data sets. (**A**) Principal components analysis (PCA) of environmental data (Euclidean distance from normalized variables). (**B–D**) Principal coordinates analysis (PCoAs) of (**B**) microbial community (16S), (**C**) broad microeukaryote community (18S raw sediment), and (**D**) meiofaunal community (18S Ludox). PCoAs used Bray-Curtis distance based on amplicon sequence variant (ASV) transformed data (i.e., relative abundance). Sample sites: Campbell Cove (tan), Westside Park (dark blue), and Mason’s Marina (pink). Habitats: bare sediment (closed circles) and seagrass (open circles).

We assessed the environmental variables of seagrass and bare sediment samples at three distinct sites at the Bodega Harbor (Fig. 1). Analysis of environmental variables grouped samples by site and habitat, and primarily highlighted differences in sediment properties (Fig. 2A). Mason’s Marina, located in the inner harbor, exhibited higher total organic carbon (TOC), silt %, and clay %, especially in seagrass habitats. Westside Park (mid harbor) and Campbell Cove (outer harbor) were characterized by higher sand % (Table S1). Differences between seagrass and bare sediment habitats varied by site, with Westside Park showing the most pronounced differences: bare sediments had higher sand %, while seagrass had higher TOC and sulfate. PC1 and PC2 together explained 99.06% of the total variation in sediment samples (Fig. 2A). Significant differences were found for all environmental variables between sites, habitats, and habitats within sites (Tables S1 and S4).

### Patterns of alpha- and beta-diversity in microbial and microeukaryote communities

We compared alpha-diversity between bare sediment and seagrass habitats within sites (Fig. S1). For microbes (16S), there were no significant differences for observed, Shannon, and Simpson diversity estimates between the two habitats (Fig. S1A). Westside Park displayed significantly higher evenness in seagrass, while Mason’s Marina had significantly higher evenness in bare sediments (Fig. S1A). For microeukaryotes (18S raw sediment), bare sediments had significantly higher observed diversity compared to seagrass at Campbell Cove and Westside Park (Fig. S1B). Additionally, bare sediments at Campbell Cove had significantly higher Shannon and Simpson diversity than seagrass (Fig. S1B). For the meiofauna data set (18S Ludox), bare sediments had higher observed diversity compared to seagrass in all three sites (Fig. S1C). At Westside Park, seagrass had significantly higher Shannon, Simpson, and evenness compared to bare sediments (Fig. S1C), while at Mason’s Marina, seagrass had significantly lower Shannon and Simpson diversity (Fig. S1C). Overall, alpha-diversity was consistently higher in Campbell Cove than in Mason’s Marina across all data sets, except for observed and Shannon in the 18S raw sediment data set (Fig. S2 and Table S3). Westside Park showed highly variable values, although it had the lowest means for all metrics in the meiofauna data set. When comparing habitats, bare sediments had higher observed and Shannon diversity in every data set. Furthermore, for microeukaryotes (18S raw sediment), bare sediment exhibited significantly higher Simpson (Table S3).

Microbial communities (16S data set) showed a strong separation by sampling sites, suggesting an environmental gradient from outer to inner sites at Bodega Harbor (Fig. 2B). Microeukaryotes (18S raw sediment) were also strongly structured by sampling sites, with slightly more overlap between Campbell Cove and Westside Park samples (Fig. 2C). Meiofaunal communities (18S Ludox) showed a similar pattern but with much more overlap among sample sites (Fig. 2D). Regardless of the data set, bare sediment communities from Westside Park strongly differed from all other samples. PERMANOVA analysis and pairwise tests detected significant differences among sites and between habitats for all data sets (Table S4).

### Taxonomic composition and differential abundance of the microbial communities and predicted gene functions (16S rRNA data set)

**Fig 3:**
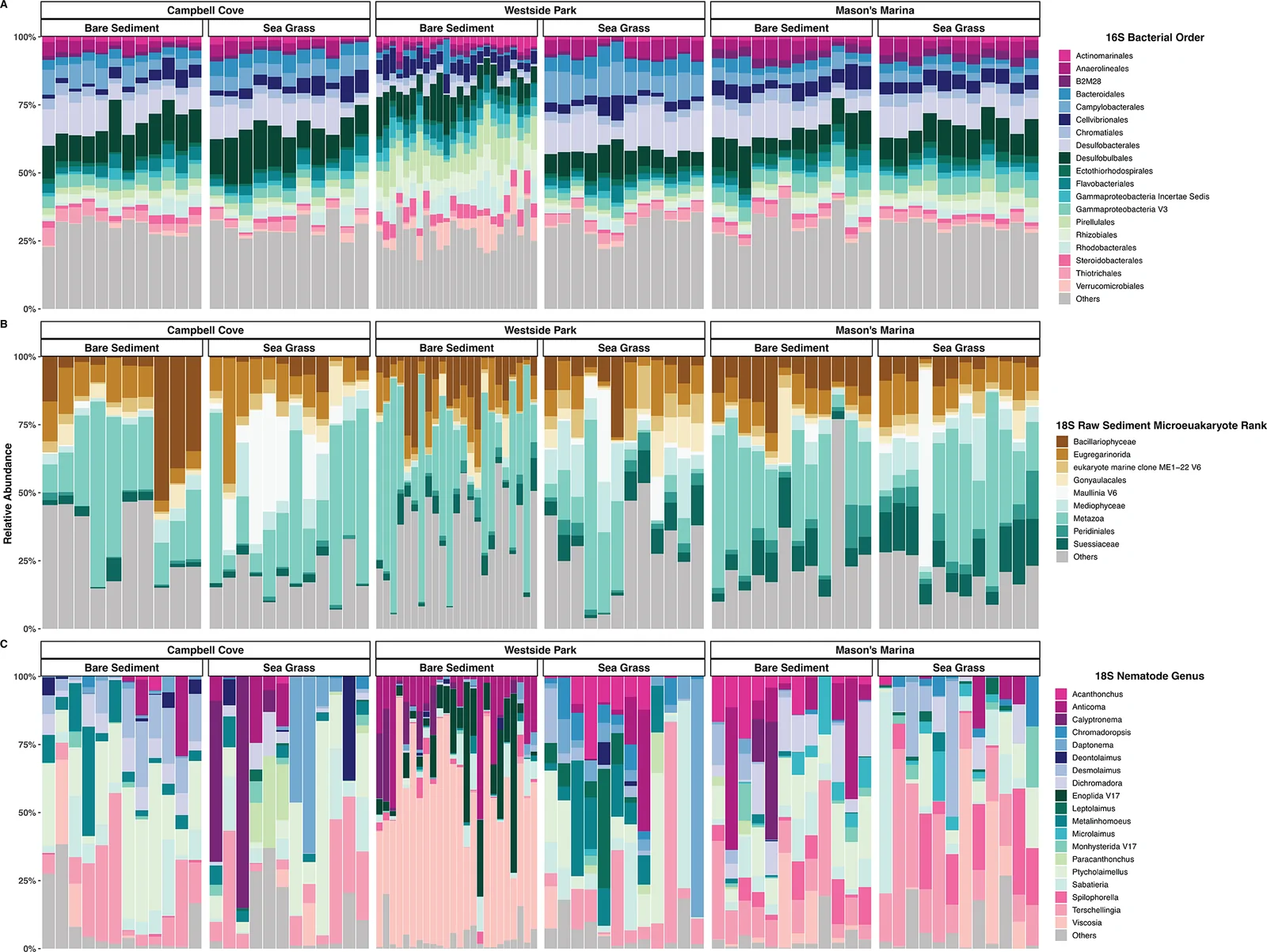
Composition of microbial, microeukaryotic, and nematode communities across sites and habitats in Bodega Harbor. (**A**) Order-level classification of the top 19 most abundant bacteria and archaea (16S). (**B**) The nine most abundant microeukaryotic taxa (18S raw sediment). (**C**) The 19 most abundant nematode genera recovered from meiofaunal samples (18S Ludox). Lower abundance taxa in each data set are grouped under “Others.”

Microbial communities were predominantly composed of the order *Desulfobulbales*, especially at Campbell Cove (Fig. 3A). *Desulfobacterales* and *Campylobacterales* were widespread, except at Westside Park bare sediments where *Pirellulales*, *Rhodobacterales*, and *Rhizobiales* were more prevalent. Among bacterial families, *Desulfocapsaceae* and *Desulfosarcinaceae* dominated across sites, though *Desulfosarcinaceae* was less common in Westside Park bare sediments (Fig. S3A). *Sulfurovaceae* was the most abundant in Westside Park seagrass, but nearly absent in Westside Park bare sediments. *Pirellulaceae* and *Rhodobacteraceae* were more abundant in Westside Park bare sediments, whereas *Halieaceae* was slightly more prevalent at Mason’s Marina.

**Fig 4:**
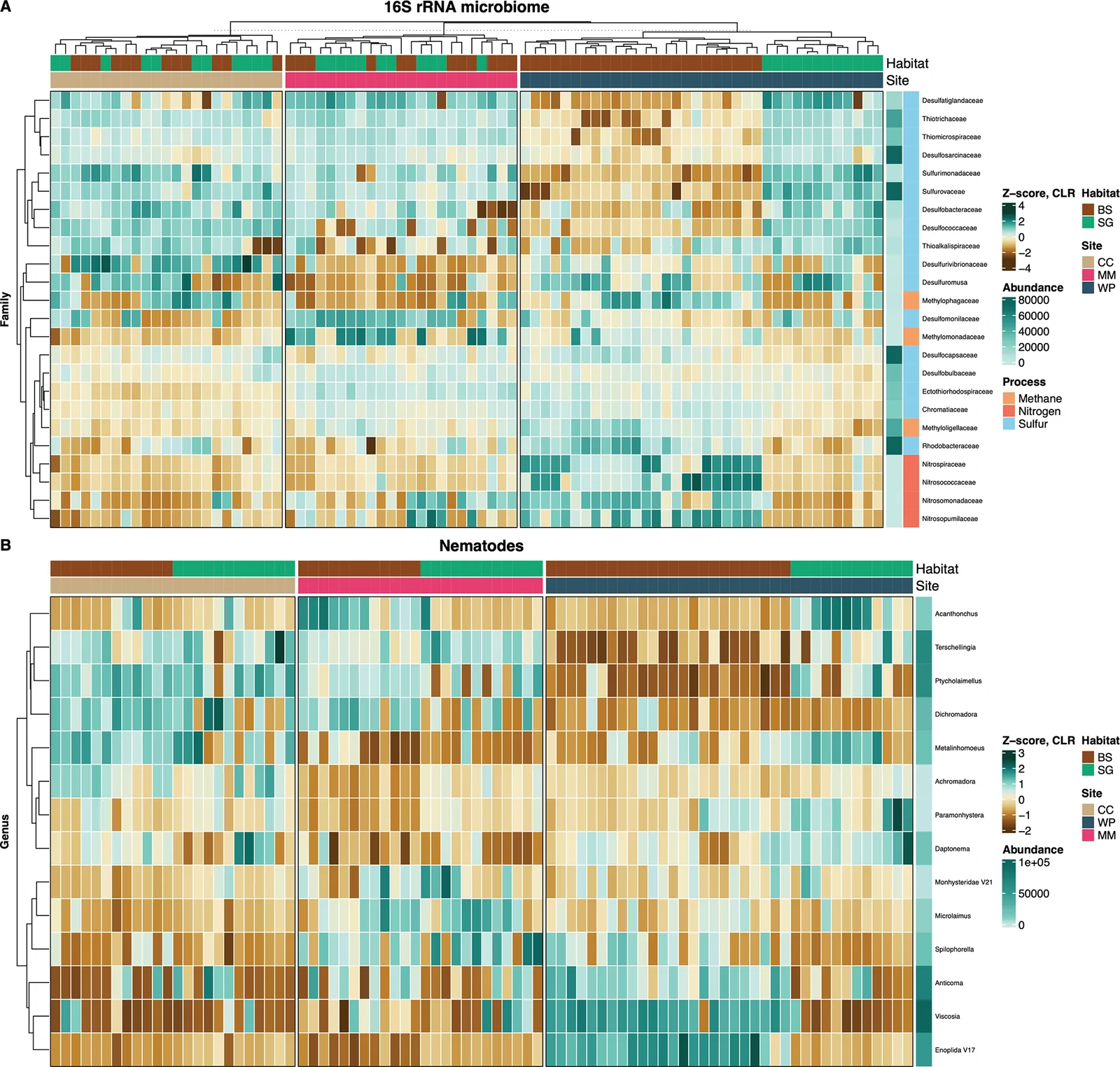
Patterns of differential abundance in bacterial/archaeal taxa (**A**) and nematode genera (**B**) across locations and habitats in Bodega Harbor. (**A**) Heatmap showing bacterial/archaeal families associated with methane, nitrogen, and sulfur cycling across sample sites. (**B**) Heatmap showing nematode genera differentially abundant in the 18S Ludox ASV data set. Sample sites (CC, Campbell Cove; WP, Westside Park; MM, Mason’s Marina) and habitats (BS, bare sediment; SG, seagrass) are abbreviated due to space constraints. Differential abundance analyses were run in the ALDEx2 software package, where ASV abundances in 16S and 18S data sets were transformed using the centered-log ratio (CLR). Dark green indicates high abundance, while brown represents low abundance. Only the most differentially abundant bacterial species are shown here; a complete list of differentially abundant bacterial genera is provided in Fig. S3.

Differential abundance analysis detected differences among bacterial taxa between sites and among habitats (Fig. 4A and Fig. S4). *Desulfocapsa* and group MSBL-7 (*Deltaproteobacteria*) were enriched at Campbell Cove, while *Sulfurimonas*, *Desulfosarcina*, and *Desulfobulbus* were depleted in Westside Park (Fig. S4). Westside Park, especially bare sediments, showed enrichment for *Anderseniella*, *Ahrensia*, *Eudoraea*, *Microbulbifer*, and *Parasphingopyxis. Halochromatium* was especially enriched at Mason’s Marina. Differentially abundant bacterial families provided insight into nutrient cycling across sites and habitats (Fig. 4A). Those involved in nitrogen (e.g., *Nitrospiraceae* and *Nitrosococcaceae*) and methane cycling were enriched in Westside Park bare sediments, as were *Methylophagaceae* at Campbell Cove and *Methylomonadaceae* at Mason’s Marina. Sulfur cycling families (e.g., *Sulfurimonadaceae* and *Desulfosarcinaceae*) were depleted in Westside Park bare sediments, whereas *Ectothiorhodospiraceae* and *Chromatiaceae* were enriched in seagrass habitats at Westside Park and Mason’s Marina.

**Fig 5:**
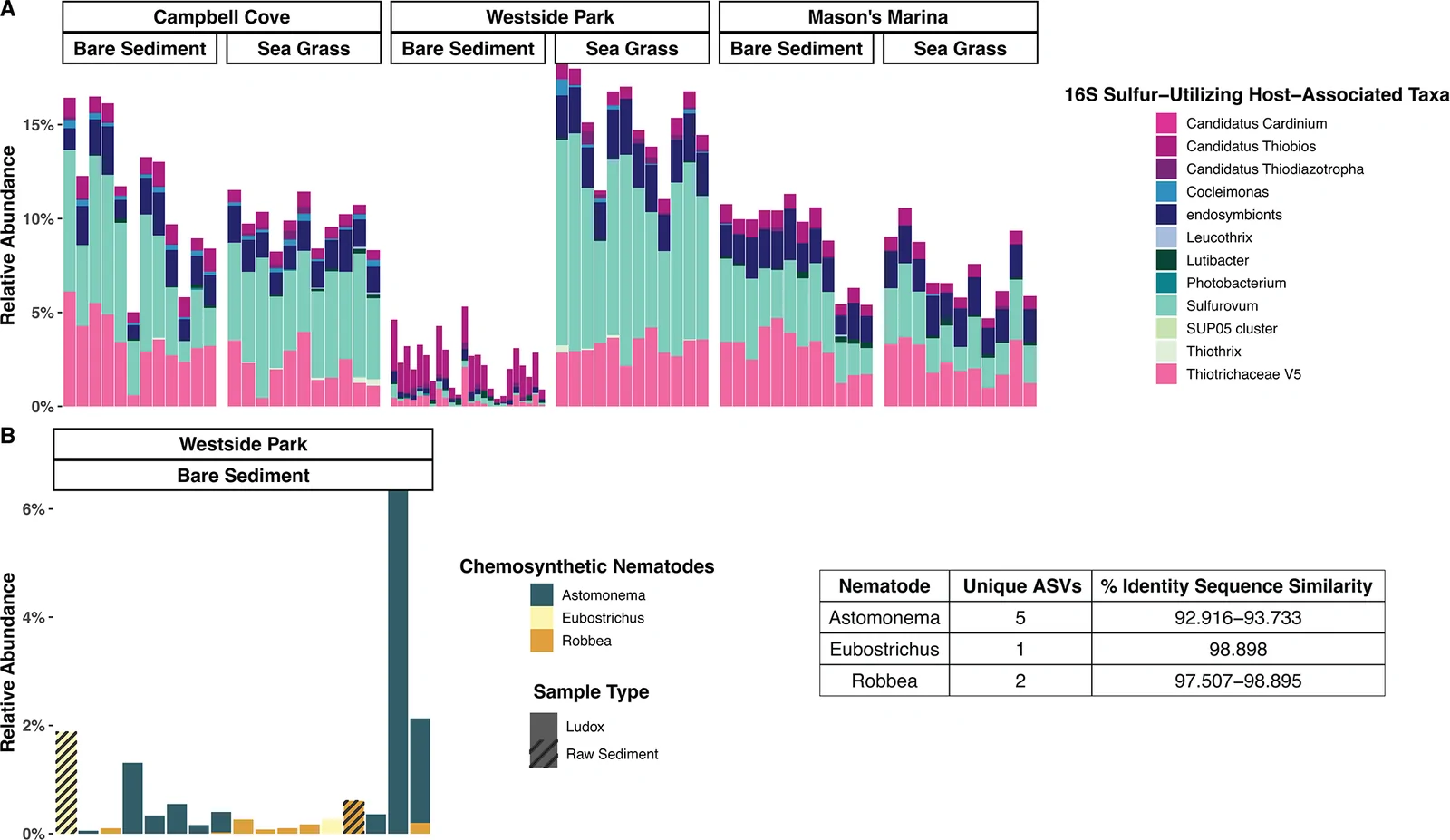
Community composition of chemosynthetic nematode taxa (*Astomonema*, *Eubostrichus*, and *Robbea*) and sulfur-utilizing, host-associated bacteria identified in eDNA data sets. Abundance bar charts show (**A**) host-associated bacterial genera known to contribute to sulfur pathways and (**B**) nematode genera within the subfamily Stilbonematinae. Bacterial genera were selected based on their involvement in sulfur pathways of interest (Fig. S4 and Table S5), as well as the literature describing symbiotic lifestyles. BLAST analysis of “Endosymbiont” sequences identified them as bacterial symbionts of *Alviniconcha*, *Thyasira*, and *Maorithyas hadalis* (all Mollusca). Nematode genera were extracted from both Ludox (15 samples) and raw sediment (2 samples) data sets. For each nematode genus, unique ASV count and percent identity were obtained from DADA2 outputs.

Overall, there were 1,618 differentially abundant predicted functions among sites, 64 related to sulfur cycling (Table S5). Pathways for dissimilatory sulfate reduction (DSR; e.g., dissimilatory sulfite reductase, adenylyl-sulfate reductase, and thiosulfate reductase) were enriched across samples, except at Westside Park bare sediments (Fig. S5). In contrast, pathways for glycosaminoglycan degradation (e.g., iduronate-2-sulfatase and N-acetylgalactosamine-4-sulfatase) were more abundant in Westside Park bare sediments. Dimethyl sulfide:cytochrome c2 reductase, a key enzyme in sulfur metabolism, was consistently elevated in Westside Park bare sediments and also enriched at Mason’s Marina. Select sulfur-associated predicted functions were used to analyze patterns in sulfur-utilizing, host-associated bacterial genera (Table S5). Westside Park bare sediments had the lowest abundances of sulfur-utilizing host-associated taxa, but the highest *Candidatus Thiobios* (an ectosymbiont of protists [39, 40]) abundance (Fig. 5A and Table S5). Sequences matching other potential endosymbionts and *Thiotrichaceae* dominated other sites and habitats.

### Major microeukaryote (18S rRNA raw sediment) and meiofauna taxa (18S rRNA Ludox)

The 18S rRNA raw sediment data set was dominated by Metazoa, though other eukaryote groups were also abundant (Fig. 3B). The parasitic protist order Eugregarinorida was notably more abundant in raw sediment samples compared to the Ludox data set. Bacillariophyceae diatoms were also commonly found in raw sediment samples, especially in bare sediment habitats. The dinoflagellate group Suessiaceae was particularly abundant in the inner estuary at Mason’s Marina, while the Phytomyxea parasite *Maullinia* was more common in seagrass habitats, especially at Campbell Cove.

Differential abundance analysis detected differences among microeukaryotes between sites and among habitats. Enriched taxa included *Gomphonema rosenstockianum* (diatom) and *Pirsonia formosa* (Stramenopiles) at Campbell Cove, dinoflagellate species *Bysmatrum subsalsum* and *Biecheleria brevisulcata* at Mason’s Marina, *Lecudina* (Alveolates), *Oblongichytrium* sp. (Stramenopiles), and DSGM-81 at Westside Park bare sediments, and *Minidiscus* sp. (diatom) at Westside Park seagrasses (Fig. S6). Metazoa also dominated the 18S Ludox reads (Fig. S3B), followed by dinoflagellate Gonyaulacales, which was widespread except at Westside Park. Fungi Dikarya were found primarily in seagrass habitats across sites, and the dinoflagellate Peridiniales was most abundant at Campbell Cove.

### Taxonomic composition of nematode communities

A total of 772 ASVs assigned to the phylum Nematoda were recovered from the 18S Ludox data set, representing 92 genera and 44 families. The most abundant families were Linhomoeidae, Chromadoridae, and Oncholaimidae (Fig. S3C). Although Anticomidae and Enchelidiidae were commonly detected, they were generally less abundant overall. Oncholaimidae, along with other putative Enoplida taxa that could not be assigned to lower taxonomic levels, was primarily present in bare sediments at Westside Park. Among genera, the most abundant were *Ptycholaimellus*, *Terschellingia*, and *Viscosia* (Fig. 3C). While *Anticoma* was frequently detected across sites, it was comparatively less abundant. *Viscosia, Calyptronema,* and Enoplida were primarily abundant in bare sediments at Westside Park. *Daptonema* and *Metalinhomoeus* were more prevalent in seagrass habitats at Campbell Cove and Westside Park.

Differential abundance analysis detected differences among nematode genera between sites and among habitats. Enriched taxa at Campbell Cove included *Achromadora, Metalinhomoeus, Terschellingia,* and *Ptycholaimellus*, with the latter two also enriched in Mason’s Marina (Fig. 4B). Genera enriched at Westside Park included *Anticoma*, Enoplida, and *Viscosia,* especially in bare sediments. Additionally, *Acanthonchus*, *Paramonhystera*, and *Daptonema* were especially enriched in Westside Park seagrass. At Mason’s Marina, unknown Monhysteridae, *Microlaimus,* and *Spilophorella* were more common. Seven unique ASV sequences identified as nematodes known to possess chemosynthetic bacterial symbionts (i.e., *Astomonema*, *Eubostrichus*, and *Robbea*) were also detected in Westside Park bare sediments (Fig. 5B). In terms of ASVs with taxonomic assignments to these known chemosynthetic nematode genera in Bodega Harbor, there were five unique ASVs assigned to *Astomonema* (92.916%–93.733% BLAST match), one *Eubostrichus* ASV (98.898% BLAST match), and two *Robbea* ASVs (97.507%–98.895% BLAST match).

### Co-occurrence network between bacteria/archaea and nematodes

**Fig 6:**
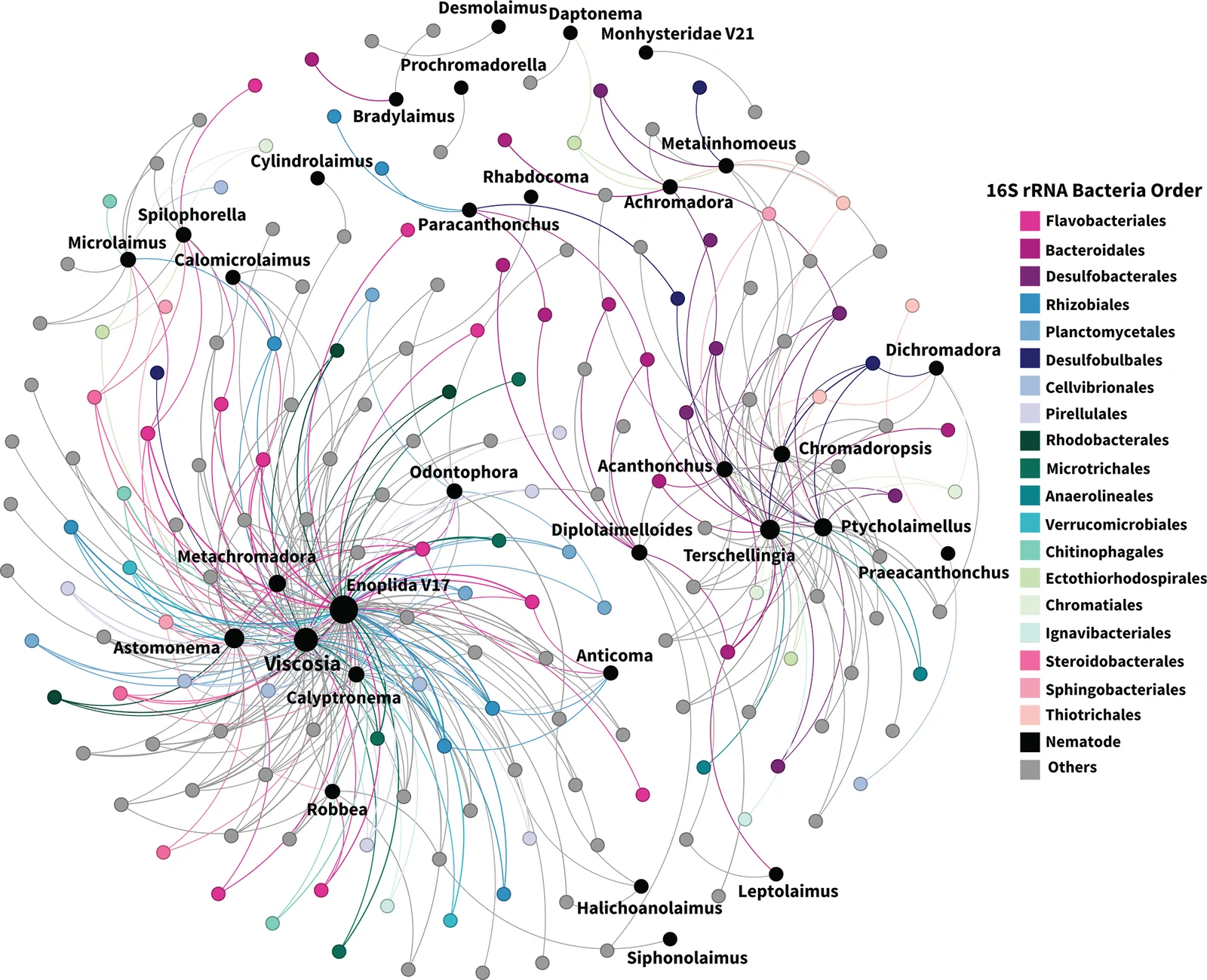
Correlation-based network analysis illustrating potential interactions between bacteria/archaea (16S, order level) and nematodes (18S Ludox, genus level). Edges (lines) between nodes indicate positive co-occurrence relationships (Spearman’s correlation coefficient [ρ] > 0.4; *P* < 0.05). Node colors distinguish nematodes (black) from various orders of bacteria/archaea; orders with fewer than three nodes are grouped as “Others.” Node size indicates the number of connections (degree) for each taxon.

The co-occurrence network, built using correlations (*r* ≥ |0.4|) and significant (*P*-value < 0.05) correlations between microbial and nematode genera, is composed of 230 nodes and 768 edges (Table S6). We focused our analysis on positive correlations to explore instances where shared environmental preferences or host-microbe relationships may exist between specific microbes and nematodes. This subnetwork contained 203 nodes and 434 edges, with 31 nodes representing nematode genera and 172 nodes corresponding to bacteria/archaea orders (Fig. 6). The network’s average degree was 4.276, with a modularity of 0.462, indicating a moderate to high level of modular structure. This suggests the presence of well-defined groups of co-occurring bacterial and nematode genera. Supporting this, a large hub in the network included multiple nematode taxa such as Enoplida, *Viscosia*, and *Astomonema*, with smaller hubs made up of one or two nematode taxa. These patterns may reflect microhabitat structures, selective associations, or environmental gradients. Overall, we identified 99 unique bacterial/archaea orders, with *Flavobacteriales* (33), *Rhizobiales* (21), *Cellvirbionales* (18), *Bacteroidales* (17), and *Desulfobacterales* (17) showing the highest degree values. Among nematode nodes, Enoplida (92) had the greatest number of connections, followed by *Viscosia* (64), *Astomonema* (37), *Terschellingia* (36), and *Ptycholaimellus* (26). Correlation coefficients for the network ranged from 0.4 (*Calomirolaimus*—d142 V3 [*Acidobacteriota*]) to 0.95 (Enoplida*—Flavobacteriales*).

## DISCUSSION

### Location and benthic environment determine community assemblages

Our eDNA study reveals a pronounced environmental gradient across Bodega Harbor, evidenced by differences in sediment composition and the grouping of benthic communities by site (outer, Campbell Cove; mid, Westside Park; inner, Mason’s Marina) rather than by habitat type (bare sediments vs seagrass beds). Environmental analyses revealed a dominance of coarse sediment (sand %) at the mouth (Campbell Cove) and midpoint (Westside Park) of Bodega Harbor, while the innermost site contained significantly higher fractions of fine sediments (silt % and clay %), and TOC (Table S1), and these sediment grain size differences explained >95% of the differences between sites within Bodega Harbor (PC1; Fig. 2A). This aligns with established estuarine dynamics: high-energy conditions near the mouth retain coarser particles, while finer sediments are transported and deposited upstream (41). Similarly, higher TOC concentrations are typically found further from estuary mouths (8, 42), and in our study, high TOC was associated with higher organic matter and reduced water flow at Mason’s Marina (43). Environmental variables also indicated the influence of benthic habitat type, with seagrass areas exhibiting higher fine sediment content, sulfate, and TOC (Fig. 2A and Table S1), likely due to lowered water velocity that limits sediment resuspension (44). Additionally, macrophyte detritus is known to enhance carbon storage and influence sulfate reduction rates, raising TOC and sulfate levels in seagrass ecosystems (31, 45, 46).

Spatial patterns for microbial, microeukaryote, and meiofaunal communities reinforce the role of site-driven environmental filtering. Microbial communities exhibited distinct shifts moving from sandy, lower-TOC sites (Campbell Cove and Westside Park) to the fine-grained, organic matter-rich sediments at Mason’s Marina, likely reflecting adaptations to nutrient and biogeochemical gradients (47), as well as community assembly based on sediment grain size (48, 49) (Fig. 2B). Microeukaryotes showed a similar trend, though there was more overlap between Campbell Cove and Westside Park seagrass assemblages (Fig. 2C). This overlap likely reflects vegetation influence (50, 51), but overall, microeukaryote communities were shaped more strongly by site differences, which aligns with prior research supporting greater similarity between habitats within the same site (8, 52). The isolation of Mason’s Marina in ordinations suggests stronger environmental filtering, likely influenced by much higher TOC and finer sediments at this location compared to all other sites.

Meiofauna assemblages showed more substantial overlap across the three sites in Bodega Harbor, and meiofaunal species were the only group to show loose clustering by bare sediment vs seagrass habitat types (Fig. 2D). Within sites, alpha-diversity patterns also often showed significant differences between bare sediment versus seagrass habitat types, particularly at Westside Park and Mason’s Marina where all four metrics were significant (Fig. S1 and Table S3), although patterns were not consistent in terms of which habitat exhibited higher biodiversity. Environmental filtering is known to be a key factor influencing the distribution of larger-body metazoan meiofaunal species such as nematodes (53, 54). In seagrass ecosystems, differences in meiofauna and nematode communities have previously been observed at multiple spatial scales, and these variations are known to be linked to increased organic matter and sediment stability from macrophytes (9, 55, 56).

Our findings indicate that benthic assemblages are primarily influenced by location within Bodega Harbor, suggesting that both prokaryotes and microeukaryotes preferentially respond to broader estuarine patterns over microhabitat variation. Sediment properties such as grain size and organic matter content are known to be the most critical environmental factors determining the composition, abundance, and diversity of meiofauna in benthic ecosystems worldwide (57–59), and our results confirm that these site-specific factors are equally important in intertidal seagrass habitats. However, microhabitat also appeared to play a secondary role in structuring benthic assemblages within Bodega Harbor. For example, microeukaryotes and meiofauna typically showed lower diversity in seagrass compared to bare sediments, although this was not always significant (Fig. S1B and C). Notably, Westside Park seagrasses—featuring higher sulfate and finer sediments—exhibited higher meiofauna diversity except for measures of richness. In estuaries, habitat effects have been shown to be strongest where environmental stress is highest, typically upstream (13). In Bodega Harbor, microeukaryote and meiofauna communities at near-mouth seagrass beds (Campbell Cove) had lower diversity than bare sediment samples; however, the difference in diversity between bare sediments and seagrass becomes less pronounced toward the inner estuary (Mason’s Marina) (Fig. S1B and C and Table S3). Taken together, our results suggest that both location and habitat specificity play a role in structuring benthic prokaryotic and microeukaryote communities, most likely governed by a complex suite of factors that include organic matter content, sulfate levels, and variations in sediment particle size and the availability of interstitial spaces among sediment particles.

### Distinction of benthic assemblages in intertidal mudflats at Westside Park

Bare sediments in Westside Park (at the midpoint of Bodega Harbor) stood out as the most distinct benthic community in both 16S and 18S metabarcoding data sets, always grouping strongly and separately from all other samples (Fig. 2B through D). These bare sediment samples were collected much farther away from seagrass beds compared to all other sites, and visual differences in this mudflat habitat are even obvious in satellite photos of Westside Park (solid blue diamond in Fig. 1A). Westside Park bare sediments were collected higher up the beach slope, and this site was characterized by mudflats exhibiting ripple marks and wave-like undulations in sediments that became exposed during low tide (Holly Bik, personal communication). In this study, bare sediments at Westside Park encompassed both “low mudflat” samples (sediment cores collected in the ripple troughs, where organic matter and detritus became concentrated) and “high mudflat” samples (sediment cores collected at the peak of a sediment ripple). This slightly farther mudflat site was chosen for coring because no clear unvegetated sediments were visible next to the Westside Park seagrass habitat during sample collection. Our initial data analysis suggested that high vs low mudflat sediment cores did not exhibit distinct benthic community assemblages, and because of this similarity, we ultimately grouped all cores into a single “bare sediments” category at Westside Park.

Our results highlight the unique features of these intertidal mudflat habitats in estuaries. Westside Park bare sediment samples had the highest sand %, and the lowest clay %, silt %, sulfate, and TOC—this granulometry somewhat resembled grain size proportions seen in Campbell Cove, but it was markedly different from geographically closer Westside Park seagrass samples (Fig. 2A and Table S1). At Westside Park bare sediments, the abiotic factors associated with tidal cycles (e.g., temperature, salinity, sediment moisture fluctuations) likely drive the observed community differences more strongly than the measured sediment properties. Previous studies have shown that intertidal mudflats display unique zonation patterns correlated with elevation and tidal exposure; for instance, higher elevated mudflats exposed to greater wave energy tend to retain more coarse sand and less fine sediment (60, 61), in line with the site characteristics seen in Westside Park bare sediment habitats. In contrast, bare sediments at Campbell Cove and Mason’s Marina, located directly adjacent to seagrass beds, may benefit from seagrass-related sediment stabilization and fine particle trapping (44), resulting in different hydrodynamic forces than at Westside Park bare sediments.

The distinction of Westside Park bare sediments is further supported by the prokaryotic and microeukaryote assemblages, which were observed in this habitat. Our ALDEx2 analyses revealed a statistically significant enrichment of ASVs with taxonomic assignments to nitrogen cycling bacteria (*Nitrospiraceae*, *Nitrosococcaceae*, *Nitrosomonadaceae*, and *Nitrosopumilaceae*) and, to a lesser extent, methane cycling bacteria (*Methylophagaceae*, *Methylomonadaceae*, and *Methyloligellaceae*) in Westside Park bare sediments (Fig. 4A). The dominance of these bacterial taxa at Westside Park is in line with previous studies showing that marine mudflats are often hotspots for microbial methanogenesis (62–64). In contrast, all other sites within Bodega Harbor—including bare sediment habitats at Campbell Cove and Mason’s Marina—showed a statistical enrichment of bacterial ASVs with taxonomic assignments to known sulfur-cycling groups, including *Desulfobacteraceae, Thiotrichaceae, and Desulfatiglandaceae* (Fig. 4A). The comparative depletion of sulfur-cycling bacterial ASVs in Westside Park bare sediments further underscores the distinct biogeochemical processes likely occurring within this intertidal mudflat habitat. This lack of sulfur-cycling pathways at Westside Park bare sediments was further supported by predicted gene functions from PICRUSt2 analyses, where DSR pathways—including thiosulfate reductase, adenylyl-sulfate reductase, and dissimilatory sulfite reductase—were highly enriched in seagrass habitats and adjacent bare sediments, but overall lacking in Westside Park bare sediments (Fig. S5). Generally, seagrass beds are known for high sulfur cycling due to oxygen production and increased organic matter content (22, 65), and the higher signal from sulfur-associated bacterial ASVs enriched in bare sediments directly adjacent to seagrasses at Campbell Cove and Mason’s Marina is more characteristic of the expected microbial metabolisms associated with seagrass ecosystems (22, 66).

Meiofauna diversity and community composition were also quite distinct within Westside Park bare sediments. Westside Park was the only site in Bodega Harbor where meiofauna assemblages in bare sediments exhibited significantly lower alpha-diversity than seagrass habitats at the same site (Fig. S1C); this pattern was significant across all four alpha-diversity metrics. At other sites, the biodiversity levels between bare sediments were not significantly different (Campbell Cove) or bare sediments showed significantly higher biodiversity across several alpha-diversity metrics (Mason’s Marina). Meiofaunal assemblages in Westside Park bare sediments were dominated by nematode ASVs with taxonomic assignments to the genus *Viscosia* and the group “Enoplida V17” (Fig. 3C and Fig. 4B). In ALDEx2 analyses examining differential abundance of ASVs, both *Viscosia* and Enoplida V17 were statistically enriched only in Westside Park bare sediments and statistically depleted at all other sites, including all other bare sediment habitats. The nematode order Enoplida (which includes the nematode genus *Viscosia* within family Oncholaimidae) is a highly diverse clade of marine nematodes and includes some of the largest-bodied marine species, many of which are facultative predators or scavengers (67, 68). *Viscosia* and other Enoplids (e.g., *Enoplolaimus litoralis*) are commonly found in sandy intertidal sediments (69–71). While these nematodes typically inhabit the upper sediment layers, they may move deeper when bioturbation occurs or in search of prey (72–74). Their swimming behavior enables rapid movement within sediments, alternating between active and inactive, which facilitates dispersal through tidal currents (75). Thus, the environmental conditions in Westside Park bare sediments—characterized by sandy sediments with larger interstitial spaces and low organic matter—may favor these larger predatory nematodes over deposit-feeding types (76, 77).

### Nematode bioindicators of environmental stress and sediment quality

More broadly, the nematode taxa recovered across all sites in Bodega Harbor support the role of sediment properties and habitat quality in structuring benthic assemblages in seagrass ecosystems. Nematodes have been touted as ideal bioindicators for monitoring environmental pollution and disturbance in aquatic ecosystems, as their small size and rapid generation time allow nematode communities to respond quickly to chemical and physical environmental stressors (59, 78), including oil spills and heavy metal contamination (79, 80). For eDNA ASVs with taxonomic assignments to Nematoda, genus-level taxonomy can be used to infer relative sensitivity or resilience to environmental perturbation, and these genus-specific nematode responses are well known and based on scientific syntheses spanning several decades of empirical studies (59).

In Bodega Harbor, nematode ASVs with taxonomic assignments to the genera *Microlaimus* and *Spilophorella* were statistically more abundant in Mason’s Marina habitats but depleted in other sites (Fig. 4B). *Microlaimus* is a commonly used nematode bioindicator, known to be able to persist in environments with hydrocarbon pollution, such as mariculture and harbors (59, 81–83). Similarly, *Spilophorella* has been previously recovered as a dominant member of the nematode community in polluted sediments across Bohai Bay, China, with the relative abundance of one *Spilophorella* sp. increasing at the most contaminated sites (84). Although we did not measure sediment contaminant levels directly in this study, these nematode bioindicators suggest that chemical pollution and hydrocarbon contamination from the nearby marina could be higher in this area of Bodega Harbor with lower water flow. ASVs with taxonomic assignments to two other nematode genera, *Terschellingia* and *Ptycholaimellus,* were found to be statistically depleted in Westside Park bare sediments (Fig. 4B) but statistically more abundant at all other sites, including all other bare sediment habitats. Notably, *Terschellingia* is resistant to anthropogenic pollution (59, 77, 85, 86), but remains sensitive to physical disturbance and harsh environments, supporting their lower abundances in Westside Park bare sediments, which are presumed to have more harsh abiotic conditions and be subjected to higher wave action and exposure during low tides. Thus, in our data set, nematode bioindicators such as *Terschellingia* appear to suggest a distinct signature of physical disturbance and abiotic stress compared to potential environmental pollutants in Bodega Harbor sediments.

The nematode genera *Metalinhomoeus* and *Daptonema* were notably enriched in Westside Park seagrass beds as well as both habitat types in Campbell Cove (Fig. 4B), but depleted in Westside Park bare sediments and largely absent from Mason’s Marina (Fig. 3C and 4B). *Daptonema* is one of the most tolerant nematode genera known, resistant to anthropogenic disturbances (59, 86–88) and typically found in environments contaminated with diesel and metals (59, 82, 87). Although *Daptonema* spp. are typically able to thrive in fine/silty sediments; experimental work has shown that they are highly sensitive to decreased oxygen levels and partially sulfidic conditions (89), which likely explains the exclusion of *Daptonema* nematodes from exposed and oxygen-depleted mudflats at Westside Park and very high sulfate conditions at Mason’s Marina. In contrast, *Metalinhomoeus* tolerates high organic matter enrichment (90) and can survive even long-term anoxia (>300 days [91]), suggesting either an unknown sensitivity of this nematode genus to high sulfide, variable species-specific sensitivity patterns, or another unknown biological or abiotic factor which may be impacting the exclusion of *Metalinhomoeus* from certain habitats in Bodega Bay.

Finally, *Achromadora* nematodes were observed to be statistically enriched in only at Campbell Cove, in both bare sediments and seagrass beds at this site. Similarly, *Dichromadora* was enriched at both habitats in Campbell Cove and Mason’s Marina, but exhibited minimal abundance overall at Westside Park seagrasses or bare sediments. The higher abundance of *Achromadora* and *Dichromadora* at these sites could not be readily explained by any obvious biological or environmental factor, although *Achromadora* is typically known as an exclusively limnetic-terrestrial nematode (92) and its presence in Campbell Cove could potentially be explained by wind transport or runoff from nearby soils. In summary, nematode bioindicator data provide further support for the sediment properties and abiotic factors structuring benthic assemblages at each site in Bodega Harbor, and provide preliminary evidence for other key factors that may be impacting benthic seagrass habitats, such as hydrocarbon pollution or hypoxic sediment conditions.

### Correlations between eukaryotes and bacteria in benthic seagrass ecosystems

In the present study, our paired 16S and 18S metabarcoding data sets allowed for a unique exploration of potential symbiotic relationships in seagrass ecosystems, both in terms of known symbioses and identification of potentially novel eukaryotic-bacterial interactions. Seagrass beds are hotspots for sulfur cycling, harboring a diversity of sulfur-utilizing bacteria and creating unique biogeochemical conditions that are known to promote symbiotic relationships between bacteria and eukaryotic hosts, including ciliate protists, gutless oligochaetes, flatworms, stilbonematid nematodes, and lucinid clams (93, 94). In each case, the eukaryotic host species harbors chemoautotrophic bacteria (either as ectosymbionts attached outside the body or endosymbionts housed within body tissues) that utilize sulfur to provide nutrition to the host, and in the case of ectosymbionts, offers additional physical protection from toxic hydrogen sulfide molecules. In many cases, the same phylogenetic clade of bacterial symbionts has independently associated with a wide variety of animal hosts. For example, *Candidatus Thiosymbion* bacteria form a coat of ectosymbiotic bacteria on the outside of the body of stilbonematid nematodes, and other species of *Ca. Thiosymbion* are endosymbionts of *Olavius* species gutless oligochaetes (94, 95).

As expected, our PICRUSt2 analyses of predicted gene functions in bacteria confirmed an abundance of sulfur-based metabolic pathways in most Bodega Harbor habitats. Predicted genes for DSR pathways—including thiosulfate reductase, adenylyl-sulfate reductase, and dissimilatory sulfite reductase—were enriched in seagrass habitats and adjacent bare sediments, except for Westside Park bare sediments (Fig. S5). The DSR pathway, which can oxidize water-insoluble sulfur particles, is a key function among symbiotic sulfur-oxidizing bacteria (96). In addition to predicted gene functions, we also utilized ASV taxonomy assignments to identify known eukaryote-associated bacterial symbionts that likely contribute to these sulfur cycles (Fig. 5A). For instance, *Candidatus Thiobios* (ectosymbiont of the marine ciliate *Zoothamnium niveum*) and *Candidatus Thiodiazotropha* (symbiont of lucinid clams such as *Loripes lucinalis*) were identified in our 16S rRNA data set and were correctly predicted as key taxa that contribute to DSR pathways in our PICRUSt2 analysis (Table S5) (96–100). Additionally, we also recovered taxa with ASV taxonomy assignments to the SUP05 cluster, including *Thiotrichaceae* (e.g., *Thiothrix*, *Cocleimonas*, and *Leucothrix*), *Sulfurovum* spp., and unclassified endosymbionts of the gastropod *Alviniconcha* and thyasirid clams *Thyasira* and *Maorithyas hadalis* (97–101). These SUP05 taxa were similarly predicted to be strong contributors to DSR pathways. Thus, our benthic eDNA approach was able to identify well-known signatures of symbiosis in seagrass ecosystems using only small volumes of sediment, without directly sampling for larger invertebrates or protistan symbioses.

We also carried out preliminary analyses to examine potential signatures of nematode-bacterial symbioses, based on the well-known occurrence of nematodes harboring endo- or ectosymbiotic bacteria in seagrass ecosystems (e.g., nematode subfamilies Stilbonematinae and Astomonematinae [94, 95]), and the common occurrence of other marine nematode genera within the nematode family Oncholaimidae where there is emerging evidence for novel bacterial symbioses with *Pseudoaltromonas* bacteria that form biofilms within the mouth, esophagus, and ovaries of host nematodes (102). Notably, ASVs assigned to nematode genera with known chemosynthetic symbionts (i.e., *Astomonema*, *Eubostrichus*, and *Robbea*) were detected only in Westside Park bare sediments (Fig. 5B). Oncholaimid nematodes, known for hosting *Pseudoalteromonas* symbionts and other as-yet-unidentified host-associated bacteria (102–104), were also statistically enriched in Westside Park bare sediments and comprised a significant proportion of the overall nematode community in this specific habitat (Fig. 3C). The higher sand content and increased interstitial spaces in Westside Park bare sediments, along with more variable sediment oxygen dynamics during tidal cycles, may offer an ideal habitat for these nematodes and their symbiont bacteria compared to other sites within Bodega Harbor (105). Thus, our data indicate that mudflat habitats within Bodega Harbor may be a hotspot for animal-bacterial symbioses, particularly for microbial metazoa such as nematodes.

Furthermore, nematodes with known symbionts were prominent in our nematode-bacterium co-occurrence network (Fig. 6). For example, the nematode genera *Viscosia* (Oncholaimidae) and *Astomonema* (Astomonematinae) exhibited 64 and 37 positive connections with bacterial ASVs, respectively (Fig. 6). Another Stilbonematid nematode genus, *Robbea,* had eight significant connections. All three nematodes shared bacterial co-occurrences with *Microbulbifer (Cellvibrionales*) and PAUC26f (*Acidobacteriota)* (Table S6). Although these bacterial ASVs do not represent the primary known *Ca. Thiosymbion* bacterial ectosymbionts in these nematode genera, we hypothesize that these statistically significant co-occurring ASVs may suggest bacterial biomarkers for suitable nematode habitat, or even potentially new co-symbionts of nematodes. *Microbulbifer*, known for degrading polysaccharides and producing broad-spectrum antibacterial alkanoyl imidazoles, is a known host-associated bacterial group that has been previously isolated from sponges and sea urchins (106–110). Similarly, PAUC26f is another sponge-associated bacterium, although its symbiotic function and evolutionary adaptations remain unknown (111). Other bacterial orders known to contain symbiotic and host-associated taxa (112) such as *Bdellovibrionales*, *Rhizobiales*, *Legionellales*, and *Polyangiales* showed notable co-occurrence patterns with nematodes from the genera *Astomonema*, *Robbea*, and *Viscosia*. One bacterium, *Muricauda* (*Flavobacteriales*), uniquely co-occurred with *Viscosia* and other Enoplid nematodes, and previous work has shown that this bacterial group is known to improve coral and clam resilience to stress via antioxidant carotenoid production (113–115).

Many additional nematode genera appeared prominently in bacterial co-occurrence networks, including almost all of the statistically significant nematode bioindicator taxa discussed above, such as nematodes from the genera *Microlaimus*, *Spilophorella*, *Terschellingia*, *Ptycholaimus*, *Daptonema*, *Metalinhomoeus*, *Achromadora*, and *Dichromadora*. Uniquely, *Microlaimus* and *Spilophorella* shared a connection with *Thiogranum* (*Ectothiorhodospirales),* a chemolithoautotrophic bacterium often linked to high concentrations of heavy metals and nutrients (116). The co-occurrence of these nematodes and *Thiogranum* suggests that these taxa may be adapted to habitats shaped by active sulfur cycling and high organic matter turnover at sites such as Mason’s Marina (81, 82, 117). Interestingly, the nematode *Diplolaimelloides* that was found only in seagrass habitats (Fig. 3C) co-occurred with *Campylobacterales* (e.g., sulfur-oxidizing genera *Sulfurimonas* and *Sulfurovum*) and *Oceanospirillales* (aerobic *Marinomonas*). In other marine invertebrate hosts, *Campylobacterales* are well-described symbionts known to possess complete sulfur-oxidizing complexes and can fix carbon dioxide via the reverse tricarboxylic acid (rTCA) and Calvin-Benson-Bassham (CBB) pathways using electrons from reduced sulfur (104, 118), also acquiring metabolites like vitamin B_6_ and molybdenum cofactor from hosts (118–121). *Oceanospirillales* bacteria are known to have symbiotic relationships with sea urchins, corals, mussels, and bone-eating worms, providing hydrocarbon degradation (122, 123). Putative nematode-bacterium associations such as *Diplolaimelloides–Oceanospirillales* may support seagrass resilience and carbon sequestration (1, 2, 4, 124). Finally, associations with sulfur-oxidizing *Sulfurimonas* and *Sulfurovum* bacteria are known to provide carbohydrates and amino acids to host invertebrates or bacterivores (100, 117, 125–127), potentially aiding environmental adaptation in sulfide-rich seagrass sediments. Further studies are needed to confirm these host-associated relationships among microscopic benthic invertebrates and investigate potential ramifications for ecosystem function in seagrass ecosystems worldwide. Bacterial symbioses in meiofaunal metazoan groups remain severely underexplored (128), with most work focusing on species with known associations with chemosynthetic bacteria (127). Symbiosis in seagrass ecosystems may be much more prevalent than currently known, and our data suggest some intriguing associations that warrant further exploration. Although we cannot draw any definitive conclusions from co-occurrence networks based on metabarcoding ASVs, these results lay the groundwork for hypothesis generation and more targeted -Omics work in future studies.

## MATERIALS AND METHODS

### Study area

Bodega Harbor, located in northern California, USA (38°19′59″N 123°02′53″W), is approximately 105 km north of San Francisco. This small low-inflow estuary (~5 km^2^) features extensive intertidal mudflats and limited fringing salt marshes. Finer sediment and higher organic content are found in the inner part of the harbor, whereas sandy sediment dominates near the harbor mouth (128). The estuary is relatively shallow (~2 m of depth at high tide) and connects to the Pacific Ocean via a dredged channel in the northern part of Bodega Harbor (129). The system is strongly affected by the adjacent ocean, with up to 80% water exchange during each tidal cycle (129, 130). Freshwater input from small streams is minimal and occurs mostly during the winter rainy season. Mean summer water temperatures range from 15.9 to 18.1°C (43, 130).

### Sampling

Sediment samples were collected at low tide from Bodega Harbor on 7 July 2018 across three different sites: Mason’s Marina (inner estuary), Westside Park (mid estuary), and Campbell Cove (outer estuary). At each site, four sediment cores were taken from vegetated (seagrass) and non-vegetated (bare sediment) habitats using a 10 × 10 cm PVC core (Fig. 1). Samples were placed in Ziploc bags, kept cool in an ice chest, and transported to the laboratory, where they were stored at −80°C until processing. Vegetated sediments at Mason’s Marina and Westside Park were covered in eelgrass (*Zostera marina*), while those at Campbell Cove also contained sea lettuce (*Ulva lactuca*). At Westside Park, no clearly unvegetated sediments were found adjacent to the seagrass habitat, so “bare sediments” here refer to both low and high mudflat samples collected at a location slightly farther away from seagrass beds. In the laboratory, sediment cores were split in half for molecular procedures and sediment analysis. Sediment characterization measured sand (sand %), silt (silt %), and clay (clay %), TOC, and sulfate sulfur (SO_4_-S). All sediment analyses were performed at the UC Davis Analytical Laboratory (Table S1). Sediment samples for molecular work were processed two weeks after collection.

### Extraction of meiofauna from sediment samples

To characterize meiofauna communities, with an emphasis on marine nematodes, sediment samples were washed using filtered artificial seawater (Instant Ocean, Spectrum Brands, Inc., USA) over a 45 μm sieve. Organisms were extracted from the sieved sediment by flotation using Ludox TM-50 (specific gravity of 1.15 [131]), following protocols described in Somerfield and Warwick (132). To minimize cross-contamination, sieves and glassware were sterilized and thoroughly washed between each core. The equipment was washed with Milli-Q ultrapure water, soaked in a 10% Sodium Metabisulfite solution (Pro Supply Outlet, CA, USA) for 1 h, and rinsed in Milli-Q ultrapure water before processing the next sample (133). The final volume in each Falcon tube was adjusted to 20 mL; a 1 mL aliquot of the meiofauna community was set aside for morphological identification and DNA barcoding of nematodes as described in Pereira et al. (134). DNA sequences are available in GenBank under accession numbers PV244184–PV244287 (135). The remaining sample in each Falcon tube was centrifuged at 5,000 rpm for 2 min to concentrate the organisms into a single pellet. After centrifugation, the supernatant was carefully discarded, and community DNA was extracted from the entire pellet (see below).

### Metabarcoding of microbial and microeukaryote communities

DNA was extracted from raw sediment (0.25 g of sediment) and Ludox-processed samples in triplicate using the ZymoBIOMICS DNA Miniprep Kit (Catalog Nos. D4300, Zymo Research Corp, Irvine, CA) according to the manufacturer’s protocol. To monitor potential kit contamination, blank controls containing water were included during the DNA extraction. Lysates were stored at −80°C until PCR amplification. For microbes, the V4 region of the 16S rRNA gene was amplified with primers 515F (5′-GTGCCAGCMGCCGCGGTAA-3′) and 806R (5′-GGACTACHVGGGTWTCTAAT-3′) (136). For the microeukaryotes (raw sediment and Ludox), a 400-bp segment of the V1–V2 hypervariable regions of the 18S rRNA gene was amplified using primers F04 (5′-GCTTGTCTCAAAGATTAAGCC-3′) and R22 (5′-GCCTGCTGCCTTCCTTGGA-3′). We chose to focus on the V1-V2 hypervariable region for the 18S rRNA metabarcoding approach because this primer set is 100% conserved in nematodes and provides recovery of >20 meiofaunal phyla, in addition to broad but patchy recovery of many fungi and protist groups (137). The V1–V2 region is thus more useful for benthic eDNA studies compared to V4 or V9, which are more commonly utilized in zooplankton and protist studies (138–140). All PCRs were performed in a sterilized laminar-flow hood. PCRs (25 µL total volume) consisted of 1 µL of DNA template, 0.5 µL of each primer (10 µM), 10 µL of Platinum Hot Start PCR Master Mix (2×) (Thermo Fisher), and 13 µL of molecular-grade water. Positive (ZymoBIOMICS Microbial Community Standard, Zymo Research, Irvine, CA) and negative controls (molecular-grade water) were included. PCR amplification followed the protocols described in Pereira et al. (134). DNA concentrations were quantified with a Qubit 3.0 fluorometer with the Qubit dsDNA HS (High Sensitivity) Assay Kit (Thermo Fisher Scientific) and normalized before library pooling. Libraries were purified using magnetic beads and size-selected (300–700 bp) on a BluePippen (Sage Science). Sequencing was carried out at the UC Davis Genomics Core on the Illumina MiSeq Platform (2 × 300 bp PE) in two runs. All wet lab protocols and bioinformatics scripts are freely available on GitHub (https://github.com/BikLab/bodega-bay-eDNA).

### Processing Illumina data using DADA2

Raw Illumina data sets were demultiplexed using a custom script to handle dual-barcode combinations. Primer sequences were trimmed with the cutadapt plugin (141). ASVs were denoised and estimated using DADA2 (142) within QIIME2 v2024.2 (143) with default parameters and by truncating forward and reverse reads at 250 bp and 200 bp, respectively. Taxonomy for 16S ASVs was assigned using the BLAST + consensus taxonomy classifier (144) with default parameters (top 10 hits, minimum confidence of 0.80, and a minimum consensus of 0.51), with the 16S rRNA SILVA 138 SSURef NR99 database as reference (145). For 18S ASVs, sequence reads were truncated at 209 bp (forward) and 230 bp (reverse), and taxonomy was assigned using the BLAST+ top hit approach (144) with a minimum confidence of 0.90 as recommended for meiofaunal communities with limited representation in reference databases (135). A custom reference data set (https://github.com/BikLab/MeioDB-18SrRNA) was used for 18S ASVs, composed of the SILVA 138 SSURef NR99 database with added short (~350 bp) and large (~700 bp) fragments covering the V1–V2 regions of the 18S rRNA retrieved from NCBI, improving the accuracy and precision of nematode taxonomy assignments (135).

### Bioinformatics and statistical analysis

Samples with low read count or suspected errors from DADA2 denoising were excluded from downstream analysis (Table S2). Potential contaminants were identified using the R package decontam v1.28 (146) with the prevalence method and a contaminant classification threshold of 0.5 (Table S2). In addition to blanks and controls, samples “MM.SG.1.3” and “CC.SG.3.1” were removed from the 16S rRNA raw sediment data set, and “CC.B.1.1.RS,” “CC.B.3.3.RS,” and “WP.MFL.2.1.1.RS” from the 18S rRNA raw sediment data set due to low read counts and suspected naming errors. For the 16S rRNA data, reads identified as mitochondrial, chloroplast, or unassigned at the domain level were removed. For the 18S rRNA data (Ludox and raw sediment), reads annotated as “humans,” unassigned, Charophyta (seagrass), and *Ulva* (green algae) were excluded to focus on microeukaryotes. Bar plots for 16S and 18S rRNA data sets were generated at multiple taxonomic ranks, and less abundant taxa were grouped as “Other.” Known sulfur-utilizing taxa with host associations were further visualized. Meiofauna and nematode diversity were analyzed in greater detail in the Ludox 18S data set, including BLAST (144) and phylogenetic placement (using SILVA ACT [147, 148]) of nematode ASVs where host genera are known to be obligately associated with chemosynthetic bacterial symbionts (*Astomonema*, *Eubostrichus*, and *Robbea*).

Quality-filtered ASV tables were used to assess the microbial and microeukaryote community compositions across habitats (seagrass and bare sediments) and sites (i.e., Campbell Cove, Westside Park, and Mason’s Marina). Alpha-diversity metrics—observed diversity, Shannon diversity *H*′ (log_2_), inverted Simpson (*D*), and Pielou’s evenness (*J’*)—were calculated using phyloseq v1.52 (149) and compared among habitats and sites. A Kruskal-Wallis (KW) test, followed by Wilcoxon pairwise analyses, was performed. The 16S rRNA sample “CC.SG.3.2” (Campbell Cove seagrass) habitat was excluded from the alpha-diversity analysis due to excessive reads (420,654) compared to the data set mean (21,256 ± 8,172). Rarefaction was not carried out for alpha-diversity analyses because it is not considered appropriate for eDNA data sets based on statistical principles (150), as rarefied counts can result in a high rate of false positives and/or discard samples that can be accurately analyzed by alternative methods. We have thus followed the recommendations of the phyloseq package developers in choosing not to rarefy data for alpha-diversity analyses.

Beta-diversity was assessed using Bray-Curtis dissimilarity and ASV relative abundances and visualized via PCoA with phyloseq (150). Permutational analysis of variance (PERMANOVA) was used to test for differences (*P*-value < 0.05) among sites and habitats. PCA using Euclidean distance matrix based on standardized environmental variables was used to characterize sites and habitats. To identify taxa (16S, bacteria; 18S raw sediment, microeukaryotes; and 18S Ludox, nematodes) that contribute most to community differences among sites and between habitats, differential abundance analyses were performed at different taxonomic ranks using the ALDEx2 v1.40 (151) after CLR transformation. Significance was assessed by KW tests (*P* < 0.05), and false discovery rates (FDRs) were estimated via the Benjamini-Hochberg (BH) procedure (152). Functional profiles of bacterial communities (16S rRNA) were predicted with PICRUSt2 v2.6.2-foss-2023a (153), with sulfur cycling/metabolism pathways analyzed separately as above.

To examine co-occurrence between bacteria (16S data set) and nematode genera (18S Ludox data set) in 82 shared samples, networks were constructed using the Spearman correlation coefficient (*r* ≥ |0.4|, *P* < 0.05 after FDR-corrected via the BH procedure) with the HMSC v.5.2-3 package. A prevalence threshold (≥5%) was applied, and taxa were agglomerated at the genus level and transformed to relative abundances. Networks were visualized with Gephi v0.10.1 (154) using the Fruchterman–Reingold algorithm (155); nodes represented bacteria (colored, labeled at the order level) and nematodes (labeled by taxon). For this study, all visualizations were created in ggplot2 v3.5.2 (156) using R version 4.5.0 (157).

## Data Availability

Raw amplicon sequences generated in this study have been deposited in the NCBI Sequence Read Archive (Bioproject: PRJNA1369552). All scripts and files required for the reproducibility of all analyses conducted in this study are freely available on GitHub (https://github.com/BikLab/bodega-bay-eDNA).

## SUPPLEMENTAL MATERIAL

Supplemental Material msphere.00431-26-s0001.pdf Supplemental figures and captions for supplemental tables.

Supplemental Tables msphere.00431-26-s0002.xlsx Tables S1-S6.

ASM does not own the copyrights to Supplemental Material that may be linked to, or accessed through, an article. The authors have granted ASM a non-exclusive, world-wide license to publish the Supplemental Material files. Please contact the corresponding author directly for reuse.

## References

[1] Duarte CM, Apostolaki ET, Serrano O, Steckbauer A, Unsworth RKF. 2025. Conserving seagrass ecosystems to meet global biodiversity and climate goals. Nat Rev Biodivers 1:150–165. doi:10.1038/s44358-025-00028-x

[2] Duffy JE. 2006. Biodiversity and the functioning of seagrass ecosystems. Mar Ecol Prog Ser 311:233–250. doi:10.3354/meps311233

[3] McKenzie LJ, Nordlund LM, Jones BL, Cullen-Unsworth LC, Roelfsema C, Unsworth RKF. 2020. The global distribution of seagrass meadows. Environ Res Lett 15:074041. doi:10.1088/1748-9326/ab7d06

[4] Duarte CM. 2002. The future of seagrass meadows. Envir Conserv 29:192–206. doi:10.1017/S0376892902000127

[5] Walter RK, Rainville EJ, O’Leary JK. 2018. Hydrodynamics in a shallow seasonally low-inflow estuary following eelgrass collapse. Estuar Coast Shelf Sci 213:160–175. doi:10.1016/j.ecss.2018.08.026

[6] Ha G, Williams SL. 2018. Eelgrass community dominated by native omnivores in Bodega Bay, California, USA. Bull Mar Sci 94:1333–1353. doi:10.5343/bms.2017.1091

[7] Horinouchi M, Tongnunui P, Kaeoprakan P, Hukiew P, Furumitsu K, Kon K, Nakamura Y, Kanou K, Yamaguchi A, Okamoto K, Sano M. 2025. Fish assemblage structures in planted seagrass habitats, compared with natural seagrass habitats: a field experiment in an intertidal bay in Trang, southern Thailand. Ichthyol Res 72:426–451. doi:10.1007/s10228-025-01012-3

[8] Alsaffar Z, Pearman JK, Cúrdia J, Ellis J, Calleja ML, Ruiz-Compean P, Roth F, Villalobos R, Jones BH, Morán XAG, Carvalho S. 2020. The role of seagrass vegetation and local environmental conditions in shaping benthic bacterial and macroinvertebrate communities in a tropical coastal lagoon. Sci Rep 10:13550. doi:10.1038/s41598-020-70318-1 PMC7419567 32782295

[9] Leduc D, Probert PK. 2011. Small-scale effect of intertidal seagrass ( *Zostera muelleri* ) on meiofaunal abundance, biomass, and nematode community structure. J Mar Biol Ass 91:579–591. doi:10.1017/S0025315410001645

[10] Gustafsson C, Hovey RK, Olsen YS, Statton J, Kendrick GA. 2025. Functional traits are moderate predictors of above‐ and below‐ground biomass in multispecies seagrass habitats. Funct Ecol 39:1522–1536. doi:10.1111/1365-2435.70058

[11] Nascimento FJA, Dahl M, Deyanova D, Lyimo LD, Bik HM, Schuelke T, Pereira TJ, Björk M, Creer S, Gullström M. 2019. Above-below surface interactions mediate effects of seagrass disturbance on meiobenthic diversity, nematode and polychaete trophic structure. Commun Biol 2:362. doi:10.1038/s42003-019-0610-4 PMC6778119 31602411

[12] Morais GC, Camargo MG, Lana P. 2016. Intertidal assemblage variation across a subtropical estuarine gradient: how good conceptual and empirical models are? Estuar Coast Shelf Sci 170:91–101. doi:10.1016/j.ecss.2015.12.020

[13] Barnes RSK. 2022. Biodiversity differentials between seagrass and adjacent bare sediment change along an estuarine gradient. Estuar Coast Shelf Sci 274:107951. doi:10.1016/j.ecss.2022.107951

[14] Fonseca G, Hutchings P, Gallucci F. 2011. Meiobenthic communities of seagrass beds (*Zostera capricorni*) and unvegetated sediments along the coast of New South Wales, Australia. Estuar Coast Shelf Sci 91:69–77. doi:10.1016/j.ecss.2010.10.003

[15] Fredriksen S, De Backer A, Boström C, Christie H. 2010. Infauna from *Zostera marina* L. meadows in Norway. Differences in vegetated and unvegetated areas. Marine Biol Res 6:189–200. doi:10.1080/17451000903042461

[16] Nguyen HM, Winters G. 2025. Trends in seagrass research in the 21st century - are we there yet? Mar Environ Res 209:107198. doi:10.1016/j.marenvres.2025.107198 40334330

[17] Brun FG, Cobo-Díaz JF, González-Ortiz V, Varela JL, Pérez-Lloréns JL, Vergara JJ. 2021. Seagrass patch complexity affects macroinfaunal community structure in intertidal areas: an in situ experiment using seagrass mimics. Diversity (Basel) 13:572. doi:10.3390/d13110572

[18] Colvin TJ, Snelgrove PVR. 2025. Role of seagrass physical structure in macrofaunal biodiversity-ecosystem functioning relationships. Mar Ecol Prog Ser 754:35–49. doi:10.3354/meps14774

[19] Boscutti F, Marcorin I, Sigura M, Bressan E, Tamberlich F, Vianello A, Casolo V. 2015. Distribution modeling of seagrasses in brackish waters of Grado-Marano lagoon (Northern Adriatic Sea). Estuar Coast Shelf Sci 164:183–193. doi:10.1016/j.ecss.2015.07.035

[20] Liu K, Guo Y, Zou M, Chen W, Hu W, Du J. 2025. Community structure and diversity of meiofauna in seagrass beds on the eastern coast of Hainan Island, China. Glob Ecol Conserv 57:e03374. doi:10.1016/j.gecco.2024.e03374

[21] Neira C, Ingels J, Mendoza G, Hernandez-Lopez E, Levin LA. 2018. Distribution of meiofauna in bathyal sediments influenced by the oxygen minimum zone off Costa Rica. Front Mar Sci 5. doi:10.3389/fmars.2018.00448

[22] Chen J, Li X, Wang H, Tang L, Xue S, Xin J, Zang Y, Tang X. 2024. The contribution of seasonal variations and *Zostera marina* presence to the bacterial community assembly of seagrass bed sediments. BMC Microbiol 24:405. doi:10.1186/s12866-024-03558-0 PMC11468120 39394553

[23] Ettinger CL, Voerman SE, Lang JM, Stachowicz JJ, Eisen JA. 2017. Microbial communities in sediment from *Zostera marina* patches, but not the *Z. marina* leaf or root microbiomes, vary in relation to distance from patch edge. PeerJ 5:e3246. doi:10.7717/peerj.3246 PMC5410140 28462046

[24] Li X, Wang H, Zang Y, Xue S, Xin J, Liu L, Tang X, Chen J. 2025. Exploring the structure and assembly of seagrass microbial communities in rhizosphere and phyllosphere. Appl Environ Microbiol 91:e02437-24. doi:10.1128/aem.02437-24 PMC11921323 39992122

[25] Cúcio C, Overmars L, Engelen AH, Muyzer G. 2018. Metagenomic analysis shows the presence of bacteria related to free-living forms of sulfur-oxidizing chemolithoautotrophic symbionts in the rhizosphere of the seagrass *Zostera marina*. Front Mar Sci 5. doi:10.3389/fmars.2018.00171

[26] Holmer M, Duarte CM, Boschker HTS, Barrón C. 2004. Carbon cycling and bacterial carbon sources in pristine and impacted Mediterranean seagrass sediments. Aquat Microb Ecol 36:227–237. doi:10.3354/ame036227

[27] Sun Y, Song Z, Zhang H, Liu P, Hu X. 2020. Seagrass vegetation affect the vertical organization of microbial communities in sediment. Mar Environ Res 162:105174. doi:10.1016/j.marenvres.2020.105174 33099080

[28] Tarquinio F, Bourgoure J, Koenders A, Laverock B, Säwström C, Hyndes GA. 2018. Microorganisms facilitate uptake of dissolved organic nitrogen by seagrass leaves. ISME J 12:2796–2800. doi:10.1038/s41396-018-0218-6 PMC6194017 29977008

[29] Pawlikowski K, Kornijów R. 2023. Above- and belowground habitat complexity created by emergent and submerged vegetation drives the structure of benthic assemblages. Oceanologia 65:358–370. doi:10.1016/j.oceano.2022.10.002

[30] Duarte B, Martins I, Rosa R, Matos AR, Roleda MY, Reusch TBH, Engelen AH, Serrão EA, Pearson GA, Marques JC, Caçador I, Duarte CM, Jueterbock A. 2018. Climate change impacts on seagrass meadows and macroalgal forests: an integrative perspective on acclimation and adaptation potential. Front Mar Sci 5. doi:10.3389/fmars.2018.00190

[31] Ugarelli K, Chakrabarti S, Laas P, Stingl U. 2017. The seagrass holobiont and its microbiome. Microorganisms 5:81. doi:10.3390/microorganisms5040081 PMC5748590 29244764

[32] Mikhailov IS, Zakharova YR, Bukin YS, Galachyants YP, Petrova DP, Sakirko MV, Likhoshway YV. 2019. Co-occurrence networks among bacteria and microbial eukaryotes of lake baikal during a spring phytoplankton bloom. Microb Ecol 77:96–109. doi:10.1007/s00248-018-1212-2 29882155

[33] Pushpakumara B, Tandon K, Willis A, Verbruggen H. 2023. Unravelling microalgal-bacterial interactions in aquatic ecosystems through 16S rRNA gene-based co-occurrence networks. Sci Rep 13:2743. doi:10.1038/s41598-023-27816-9 PMC9935533 36797257

[34] Wu J, Zhu Z, Waniek JJ, Niu M, Wang Y, Zhang Z, Zhou M, Zhang R. 2023. The biogeography and co-occurrence network patterns of bacteria and microeukaryotes in the estuarine and coastal waters. Mar Environ Res 184:105873. doi:10.1016/j.marenvres.2023.105873 36628821

[35] Bengtsson MM, Bühler A, Brauer A, Dahlke S, Schubert H, Blindow I. 2017. Eelgrass leaf surface microbiomes are locally variable and highly correlated with epibiotic eukaryotes. Front Microbiol 8:1312. doi:10.3389/fmicb.2017.01312 PMC5507959 28751881

[36] Turon X, Zarcero J, Antich A, Wangensteen OS, Ballesteros E, Cebrian E, Marco-Méndez C, Alcoverro T. 2023. Metabarcoding the eukaryotic community of a threatened, iconic Mediterranean habitat: *Posidonia oceanica* seagrass meadows. Front Mar Sci 10. doi:10.3389/fmars.2023.1145883

[37] Sun H, Wang T, Liu S, Tang X, Sun J, Liu X, Zhao Y, Shen P, Zhang Y. 2024. Novel insights into the rhizosphere and seawater microbiome of *Zostera marina* in diverse mariculture zones. Microbiome 12:27. doi:10.1186/s40168-024-01759-3 PMC10865565 38350953

[38] Bik HM, Porazinska DL, Creer S, Caporaso JG, Knight R, Thomas WK. 2012. Sequencing our way towards understanding global eukaryotic biodiversity. Trends Ecol Evol 27:233–243. doi:10.1016/j.tree.2011.11.010 PMC3311718 22244672

[39] Rinke C, Schmitz-Esser S, Stoecker K, Nussbaumer AD, Molnár DA, Vanura K, Wagner M, Horn M, Ott JA, Bright M. 2006. “Candidatus Thiobios zoothamnicoli,” an ectosymbiotic bacterium covering the giant marine ciliate *Zoothamnium niveum*. Appl Environ Microbiol 72:2014–2021. doi:10.1128/AEM.72.3.2014-2021.2006 PMC1393213 16517650

[40] Petersen JM, Kemper A, Gruber-Vodicka H, Cardini U, van der Geest M, Kleiner M, Bulgheresi S, Mußmann M, Herbold C, Seah BKB, Antony CP, Liu D, Belitz A, Weber M. 2016. Chemosynthetic symbionts of marine invertebrate animals are capable of nitrogen fixation. Nat Microbiol 2:16195. doi:10.1038/nmicrobiol.2016.195 PMC6872982 27775707

[41] Chilton D, Hamilton DP, Nagelkerken I, Cook P, Hipsey MR, Reid R, Sheaves M, Waltham NJ, Brookes J. 2021. Environmental flow requirements of estuaries: providing resilience to current and future climate and direct anthropogenic changes. Front Environ Sci 9. doi:10.3389/fenvs.2021.764218

[42] Wang C, Ding Y, Guo Z, Lin H, Wu J. 2022. Spatial–temporal distribution of total organic carbon and its transportation in the Jiulong River Estuary. Sci Rep 12:9395. doi:10.1038/s41598-022-13268-0 PMC9174163 35672394

[43] Kardish MR, Stachowicz JJ. 2023. Local environment drives rapid shifts in composition and phylogenetic clustering of seagrass microbiomes. Sci Rep 13:3673. doi:10.1038/s41598-023-30194-x PMC9985655 36871071

[44] Buonassissi CJ, Dierssen HM. 2010. A regional comparison of particle size distributions and the power law approximation in oceanic and estuarine surface waters. J Geophys Res 115. doi:10.1029/2010JC006256

[45] Kim B, Choi A, Lee K-S, Kang C-K, Hyun J-H. 2017. Sulfate reduction and sulfur cycles at two seagrass beds inhabited by cold affinity *Zostera marina* and warm affinity *Halophila nipponica* in temperate coastal waters. Estuar Coasts 40:1346–1357. doi:10.1007/s12237-017-0225-2

[46] Lee H, Morrison C, Doriean NJC, Welsh DT, Bennett WW. 2024. Trace metal distribution in seagrass-vegetated sediments of an urbanized estuary in Queensland, Australia. Mar Pollut Bull 208:116981. doi:10.1016/j.marpolbul.2024.116981 39299194

[47] Yi J, Lo LSH, Cheng J. 2020. Dynamics of microbial community structure and ecological functions in estuarine intertidal sediments. Front Mar Sci 7. doi:10.3389/fmars.2020.585970

[48] Sharma SS, Alfaro AC, Campbell KA. 2022. Influence of habitat on meiofaunal abundance and distribution in a New Zealand temperate estuary. N Z J Mar Freshw Res 56:107–134. doi:10.1080/00288330.2020.1865415

[49] Alves AS, Adão H, Ferrero TJ, Marques JC, Costa MJ, Patrício J. 2013. Benthic meiofauna as indicator of ecological changes in estuarine ecosystems: the use of nematodes in ecological quality assessment. Ecol Indic 24:462–475. doi:10.1016/j.ecolind.2012.07.013

[50] Kindeberg T, Severinson J, Carlsson P. 2022. Eelgrass meadows harbor more macrofaunal species but bare sediments can be as functionally diverse. J Exp Mar Biol Ecol 554:151777. doi:10.1016/j.jembe.2022.151777

[51] Pan Y, Li G, Su L, Zheng P, Wang Y, Shen Z, Chen Z, Han Q, Gong J. 2022. Seagrass colonization alters diversity, abundance, taxonomic, and functional community structure of benthic microbial eukaryotes. Front Microbiol 13:901741. doi:10.3389/fmicb.2022.901741 PMC9234489 35770161

[52] Barnes RSK, Barnes MKS. 2012. Shore height and differentials between macrobenthic assemblages in vegetated and unvegetated areas of an intertidal sandflat. Estuar Coast Shelf Sci 106:112–120. doi:10.1016/j.ecss.2012.05.011

[53] Luan L, Jiang Y, Cheng M, Dini-Andreote F, Sui Y, Xu Q, Geisen S, Sun B. 2020. Organism body size structures the soil microbial and nematode community assembly at a continental and global scale. Nat Commun 11:6406. doi:10.1038/s41467-020-20271-4 PMC7747634 33335105

[54] Wang W, Sun Z, Mishra S, Xia S, Lin L, Yang X. 2023. Body size determines multitrophic soil microbiota community assembly associated with soil and plant attributes in a tropical seasonal rainforest. Mol Ecol 32:6294–6303. doi:10.1111/mec.16585 35770463

[55] De Troch M, Gurdebeke S, Fiers F, Vincx M. 2001. Zonation and structuring factors of meiofauna communities in a tropical seagrass bed (Gazi Bay, Kenya). J Sea Res 45:45–61. doi:10.1016/S1385-1101(00)00055-1

[56] Liao J-X, Yeh H-M, Mok H-K. 2015. Meiofaunal communities in a tropical seagrass bed and adjacent unvegetated sediments with note on sufficient sample size for determining local diversity indices. Zool Stud 54:e14. doi:10.1186/s40555-014-0101-8 PMC6661371 31966101

[57] Kim H-G, Yoon SJ, Lee J, Hong S, Kwon B-O, Hu W, Wang T, Khim JS. 2025. Distribution pattern of meiofauna assemblages along an environment gradient on the coast of the Yellow and Bohai Seas. Mar Pollut Bull 216:117966. doi:10.1016/j.marpolbul.2025.117966 40253968

[58] Wang X, Liu X, Xu J. 2019. Distribution patterns of meiofauna assemblages and their relationship with environmental factors of deep sea adjacent to the yap trench, western Pacific Ocean. Front Mar Sci 6. doi:10.3389/fmars.2019.00735

[59] Ridall A, Ingels J. 2021. Suitability of free-living marine nematodes as bioindicators: status and future considerations. Front Mar Sci 8. doi:10.3389/fmars.2021.685327

[60] Dyer KR, Christie MC, Wright EW. 2000. The classification of intertidal mudflats. Cont Shelf Res 20:1039–1060. doi:10.1016/S0278-4343(00)00011-X

[61] Green MO, Coco G. 2014. Review of wave-driven sediment resuspension and transport in estuaries. Rev Geophys 52:77–117. doi:10.1002/2013RG000437

[62] Zhang Y, Li Q, Dai Q, Kang Y. 2019. Microbial mechanism underlying high and stable methane oxidation rates during mudflat reclamation with long-term rice cultivation: Illumina high-throughput sequencing-based data analysis. J Hazard Mater 371:332–341. doi:10.1016/j.jhazmat.2019.03.032 30856444

[63] Narrowe AB, Borton MA, Hoyt DW, Smith GJ, Daly RA, Angle JC, Eder EK, Wong AR, Wolfe RA, Pappas A, Bohrer G, Miller CS, Wrighton KC. 2019. Uncovering the diversity and activity of methylotrophic methanogens in freshwater wetland soils. mSystems 4:e00320-19 doi:10.1128/mSystems.00320-19 PMC6890927 31796563

[64] Dong W, Zhou J, Zhang C-J, Yang Q, Li M. 2024. Methylotrophic substrates stimulated higher methane production than competitive substrates in mangrove sediments. Sci Total Environ 951:175677. doi:10.1016/j.scitotenv.2024.175677 39181265

[65] Azzoni R, Giordani G, Bartoli M, Welsh DT, Viaroli P. 2001. Iron, sulphur and phosphorus cycling in the rhizosphere sediments of a eutrophic *Ruppia cirrhosa* meadow (Valle Smarlacca, Italy). J Sea Res 45:15–26. doi:10.1016/S1385-1101(00)00056-3

[66] Cúcio C, Engelen AH, Costa R, Muyzer G. 2016. Rhizosphere microbiomes of European + seagrasses are selected by the plant, but are not species specific. Front Microbiol 7:440. doi:10.3389/fmicb.2016.00440 PMC4815253 27065991

[67] Platonova TA, Galtsova VV. 1985. Nematodes and their role in the meiobenthos. Amerind Publishing Co, New Delhi.

[68] Bik HM, Lambshead PJD, Thomas WK, Lunt DH. 2010. Moving towards a complete molecular framework of the Nematoda: a focus on the Enoplida and early-branching clades. BMC Evol Biol 10:353. doi:10.1186/1471-2148-10-353 PMC2995457 21073704

[69] Pereira TJ, Fonseca G, Mundo-Ocampo M, Guilherme BC, Rocha-Olivares A. 2010. Diversity of free-living marine nematodes (Enoplida) from Baja California assessed by integrative taxonomy. Mar Biol 157:1665–1678. doi:10.1007/s00227-010-1439-z PMC3873033 24391248

[70] Sun L, Gu H, Huang Y. 2025. Two new species of the genus *Viscosia* de Man, 1890 (Nematoda, Enoplea, Enoplida, Oncholaimidae) from the intertidal zone of the Yellow Sea, China. Zookeys 1231:99–117. doi:10.3897/zookeys.1231.142078 PMC11920823 40109895

[71] Samad S, Mohammad M, Salleh S, Darif A. 2018. A checklist of free-living marine nematodes at different ecosystem in northern straits of Malacca, Malaysia. Scri Biol 5:1. doi:10.20884/1.sb.2018.5.1.667

[72] Vranken G, Vincx M, Heip C. 1985. The ecology of marine nematodes. Ocean Mar Biol 23:399–489.

[73] Pinto TK, Austen MC, Bemvenuti CE. 2006. Effects of macroinfauna sediment disturbance on nematode vertical distribution. J Mar Biol Ass 86:227–233. doi:10.1017/S0025315406013075

[74] Maria TF, Vanaverbeke J, Esteves AM, De Troch M, Vanreusel A. 2012. The importance of biological interactions for the vertical distribution of nematodes in a temperate ultra-dissipative sandy beach. Estuar Coast Shelf Sci 97:114–126. doi:10.1016/j.ecss.2011.11.030

[75] Thomas MC, Lana PC. 2011. A new look into the small-scale dispersal of free-living marine nematodes. Zoologia (Curitiba) 28:449–456. doi:10.1590/S1984-46702011000400006

[76] Montagna PA, Li J. 1997. Modelling contaminant effects on deposit feeding nematodes near Gulf of Mexico production platforms. Ecol Modell 98:151–162. doi:10.1016/S0304-3800(96)01911-4

[77] Gyedu-Ababio TK, Furstenberg JP, Baird D, Vanreusel A. 1999. Nematodes as indicators of pollution: a case study from the Swartkops River system, South Africa. Hydrobiologia 397:155–169. doi:10.1023/A:1003617825985

[78] Schratzberger M, Somerfield PJ. 2020. Effects of widespread human disturbances in the marine environment suggest a new agenda for meiofauna research is needed. Sci Total Environ 728:138435. doi:10.1016/j.scitotenv.2020.138435 32570325

[79] Semprucci F, Losi V, Moreno M. 2015. A review of Italian research on free-living marine nematodes and the future perspectives on their use as ecological indicators (EcoInds). Medit Mar Sci 16:352. doi:10.12681/mms.1072

[80] Balsamo M, Semprucci F, Frontalini F, Coccioni R. 2012. Meiofauna as a tool for marine ecosystem biomonitoring. In Cruzado A (ed), Marine ecosystems. InTech.

[81] Moreno M, Vezzulli L, Marin V, Laconi P, Albertelli G, Fabiano M. 2008. The use of meiofauna diversity as an indicator of pollution in harbours. ICES J Mar Sci 65:1428–1435. doi:10.1093/icesjms/fsn116

[5] Ng W-L, Chen C-A, Mustafa S, Soo C-L, Liao Y-C, Shih T-W. 2022 Free-living marine nematodes community structure in the conservation area (Chaojing Park) and its adjacent area of Keelung, Taiwan. PLoS One 17:e0268691. doi:10.1371/journal.pone.0268691 PMC9140311 35622785

[83] Nanajkar M, Ingole BS. 2007. Nematode species diversity as indicator of stressed benthic environment along the central west coast of India. Goa University, India. Available from: http://drs.nio.org/drs/handle/2264/680. Retrieved 26 Sep 2025.

[84] Hua E, Zhu Y, Huang D, Liu X. 2021. Are free-living nematodes effective environmental quality indicators? Insights from Bohai Bay, China. Ecol Indic 127:107756. doi:10.1016/j.ecolind.2021.107756

[85] Soko MI, Gyedu-Ababio TK. 2019. Free-living nematodes as pollution indicator in incomati River Estuary, Mozambique. OJE 09:117–133. doi:10.4236/oje.2019.95010

[86] Shokoohi E, Khan MR, Handoo Z. 2025. Nematodes as bioindicators of heavy metal contamination and soil salinity, p 298–315. In Nematode disease complexes in agricultural crops. CABI, GB.

[87] Somerfield P, Gee J, Warwick R. 1994. Soft sediment meiofaunal community structure in relation to a long-term heavy metal gradient in the Fal estuary system. Mar Ecol Prog Ser 105:79–88. doi:10.3354/meps105079

[88] Sroczyńska K, Chainho P, Vieira S, Adão H. 2021. What makes a better indicator? Taxonomic vs functional response of nematodes to estuarine gradient. Ecol Indic 121:107113. doi:10.1016/j.ecolind.2020.107113

[89] Steyaert M, Moodley L, Nadong T, Moens T, Soetaert K, Vincx M. 2007. Responses of intertidal nematodes to short-term anoxic events. J Exp Mar Biol Ecol 345:175–184. doi:10.1016/j.jembe.2007.03.001

[90] Ferreira RC, Nascimento-Junior AB, Santos PJP, Botter-Carvalho ML, Pinto TK. 2015. Responses of estuarine nematodes to an increase in nutrient supply: an in situ continuous addition experiment. Mar Pollut Bull 90:115–120. doi:10.1016/j.marpolbul.2014.11.012 25499965

[91] Taheri M, Grego M, Riedel B, Vincx M, Vanaverbeke J. 2015. Patterns in nematode community during and after experimentally induced anoxia in the northern Adriatic Sea. Mar Environ Res 110:110–123. doi:10.1016/j.marenvres.2015.08.004 26322855

[92] Holovachov O, Schmidt-Rhaesa A. 2014. Handbook of zoology. gastrotricha, cycloneuralia, gnathifera. In Nematoda. Vol. 2. de Gruyter.

[93] van der Heide T, Govers LL, de Fouw J, Olff H, van der Geest M, van Katwijk MM, Piersma T, van de Koppel J, Silliman BR, Smolders AJP, van Gils JA. 2012. A three-stage symbiosis forms the foundation of seagrass ecosystems. Science 336:1432–1434. doi:10.1126/science.1219973 22700927

[94] Sogin EM, Leisch N, Dubilier N. 2020. Chemosynthetic symbioses. Curr Biol 30:R1137–R1142. doi:10.1016/j.cub.2020.07.050 33022256

[95] Sogin EM, Kleiner M, Borowski C, Gruber-Vodicka HR, Dubilier N. 2021. Life in the dark: phylogenetic and physiological diversity of chemosynthetic symbioses. Annu Rev Microbiol 75:695–718. doi:10.1146/annurev-micro-051021-123130 34351792

[96] Sudo M, Osvatic J, Taylor JD, Dufour SC, Prathep A, Wilkins LGE, Rattei T, Yuen B, Petersen JM. 2024. *SoxY* gene family expansion underpins adaptation to diverse hosts and environments in symbiotic sulfide oxidizers. mSystems 9:e01135-23. doi:10.1128/msystems.01135-23 PMC11237559 38747602

[97] Bland JA, Brock TD. 1973. The marine bacterium *Leucothrix mucor* as an algal epiphyte. Mar Biol 23:283–292. doi:10.1007/BF00389335

[98] Brigmon RL De Ridder C 1998. Symbiotic relationship of thiothrix spp. with an echinoderm. Appl Environ Microbiol 64:3491–3495. doi:10.1128/AEM.64.9.3491-3495.1998 PMC106752 9726902

[99] Zhou K, Zhang R, Sun J, Zhang W, Tian R-M, Chen C, Kawagucci S, Xu Y. 2019. Potential interactions between clade SUP05 sulfur-oxidizing bacteria and phages in hydrothermal vent sponges. Appl Environ Microbiol 85:e00992-19. doi:10.1128/AEM.00992-19 PMC6821954 31492669

[100] Lee W-K, Juniper SK, Perez M, Ju S-J, Kim S-J. 2021. Diversity and characterization of bacterial communities of five co‐occurring species at a hydrothermal vent on the Tonga Arc. Ecol Evol 11:4481–4493. doi:10.1002/ece3.7343 PMC8093707 33976824

[101] Liu J, Zhou Y, Feng J, Cai C, Zhang S. 2025. Comparative metagenomic analysis reveals the adaptive evolutionary traits of siboglinid tubeworm symbionts. Front Microbiol 16:1533506. doi:10.3389/fmicb.2025.1533506 PMC12045306 40313410

[102] De Santiago A, Barnes S, Pereira TJ, Marcellino Barros M, Durden L, Han MK, Thrash JC, Bik HM. 2026. Pseudoalteromonas is a symbiont of marine invertebrates that exhibits broad patterns of phylosymbiosis. ISME J 20:wrag091. doi:10.1093/ismejo/wrag091 PMC13245728 42007585

[103] Zeppilli D, Bellec L, Cambon-Bonavita M-A, Decraemer W, Fontaneto D, Fuchs S, Gayet N, Mandon P, Michel LN, Portail M, Smol N, Sørensen MV, Vanreusel A, Sarrazin J. 2019. Ecology and trophic role of *Oncholaimus dyvae sp. nov.* (Nematoda: *Oncholaimidae*) from the lucky strike hydrothermal vent field (Mid-Atlantic Ridge). BMC Zool 4. doi:10.1186/s40850-019-0044-y

[104] Bellec L, Bonavita M-A, Hourdez S, Jebbar M, Tasiemski A, Durand L, Gayet N, Zeppilli D. 2019. Chemosynthetic ectosymbionts associated with a shallow-water marine nematode. Sci Rep 9:7019. doi:10.1038/s41598-019-43517-8 PMC6505526 31065037

[105] Bayer C, Heindl NR, Rinke C, Lücker S, Ott JA, Bulgheresi S. 2009. Molecular characterization of the symbionts associated with marine nematodes of the genus Robbea. Environ Microbiol Rep 1:136–144. doi:10.1111/j.1758-2229.2009.00019.x PMC2761003 19838308

[106] Jeong SH, Yang S-H, Jin HM, Kim JM, Kwon KK, Jeon CO. 2013. *Microbulbifer gwangyangensis sp.* nov. and *Microbulbifer pacificus sp*. nov., isolated from marine environments. Int J Syst Evol Microbiol 63:1335–1341. doi:10.1099/ijs.0.042606-0 22798656

[107] Lee J-Y, Kim PS, Hyun D-W, Kim HS, Shin N-R, Jung M-J, Yun J-H, Kim M-S, Whon TW, Bae J-W. 2017. *Microbulbifer echini sp.* nov., isolated from the gastrointestinal tract of a purple sea urchin, *Heliocidaris crassispina*. Int J Syst Evol Microbiol 67:998–1004. doi:10.1099/ijsem.0.001731 27959777

[108] Nishijima M, Takadera T, Imamura N, Kasai H, An K-D, Adachi K, Nagao T, Sano H, Yamasato K. 2009. *Microbulbifer variabilis sp*. nov. and *Microbulbifer epialgicus sp.* nov., isolated from Pacific marine algae, possess a rod-coccus cell cycle in association with the growth phase. Int J Syst Evol Microbiol 59:1696–1707. doi:10.1099/ijs.0.006452-0 19542114

[109] Karim MRU, Harunari E, Oku N, Akasaka K, Igarashi Y. 2020. Bulbimidazoles A-C, antimicrobial and cytotoxic alkanoyl imidazoles from a marine gammaproteobacterium *Microbulbifer* Species. J Nat Prod 83:1295–1299. doi:10.1021/acs.jnatprod.0c00082 32191468

[110] Huang H, Mo K, Hu Y, Liu M, Zhu J, Zou X, Bao S. 2020. *Microbulbifer harenosus* sp. nov., an alginate-degrading bacterium isolated from coastal sand. Int J Syst Evol Microbiol 70:1639–1643. doi:10.1099/ijsem.0.003945 32125262

[111] Indraningrat AAG, Steinert G, Becking LE, Mueller B, de Goeij JM, Smidt H, Sipkema D. 2022. Sponge holobionts shift their prokaryotic communities and antimicrobial activity from shallow to lower mesophotic depths. Antonie Van Leeuwenhoek 115:1265–1283. doi:10.1007/s10482-022-01770-4 PMC9534810 35998007

[112] Villada JC, Vasquez YM, Szabo G, Whittaker-Walker E, Romero MF, Qin S, Varghese N, Eloe-Fadrosh EA, Kyrpides NC, Visel A, Woyke T, Schulz F, SymGs data consortium. 2025. A genomic catalog of Earth’s bacterial and archaeal symbionts. bioRxiv. doi:10.1101/2025.05.29.656868 42675165

[113] Motone K, Takagi T, Aburaya S, Miura N, Aoki W, Ueda M. 2020. A zeaxanthin-producing bacterium isolated from the algal phycosphere protects coral endosymbionts from environmental stress. mBio 11:e01019-19. doi:10.1128/mBio.01019-19 PMC6974559 31964724

[114] Matthews JL, Khalil A, Siboni N, Bougoure J, Guagliardo P, Kuzhiumparambil U, DeMaere M, Le Reun NM, Seymour JR, Suggett DJ, Raina J-B. 2023. Coral endosymbiont growth is enhanced by metabolic interactions with bacteria. Nat Commun 14:6864. doi:10.1038/s41467-023-42663-y PMC10611727 37891154

[115] Uchida T, Li Y, Yamashita H, Shimada G, Shinzato C. 2025. Microbiome of the boring giant clam provides insights into holobiont resilience under coral reef environmental stress. Environ Microbiol 27:e70161. doi:10.1111/1462-2920.70161 PMC12326292 40765519

[116] Birrer SC, Wemheuer F, Dafforn KA, Gribben PE, Steinberg PD, Simpson SL, Potts J, Scanes P, Doblin MA, Johnston EL. 2021. Legacy metal contaminants and excess nutrients in low flow estuarine embayments alter composition and function of benthic bacterial communities. Front Microbiol 12:661177. doi:10.3389/fmicb.2021.661177 PMC8531495 34690940

[117] Mou A, Li X, Li Z, Qu L, Dong Y, Wang Z, Zhang X, Xu Q. 2025. Comparative analysis of esophageal gland microbes between two body sizes of *Gigantopelta aegis*, a hydrothermal snail from the Southwest Indian Ridge. Microbiol Spectr 13:e02959-24. doi:10.1128/spectrum.02959-24 PMC11960433 39992146

[118] Xu Z, Wang M, Zhang H, He W, Cao L, Lian C, Zhong Z, Wang H, Fu L, Zhang X, Li C. 2022. Metabolism interactions promote the overall functioning of the episymbiotic chemosynthetic community of *Shinkaia crosnieri* of Cold Seeps. mSystems 7:e00320-22. doi:10.1128/msystems.00320-22 PMC9426478 35938718

[119] Warnhoff K, Ruvkun G. 2019. Molybdenum cofactor transfer from bacteria to nematode mediates sulfite detoxification. Nat Chem Biol 15:480–488. doi:10.1038/s41589-019-0249-y PMC6470025 30911177

[120] Zečić A, Dhondt I, Braeckman BP. 2019. The nutritional requirements of *Caenorhabditis elegans*. Genes Nutr 14:15. doi:10.1186/s12263-019-0637-7 PMC6501307 31080524

[121] Ferreira J. 2024. Vitamin B6 is essential for *C. elegans* development. Lab Anim 53:113–113. doi:10.1038/s41684-024-01375-6

[122] Cao Y, Chastain RA, Eloe EA, Nogi Y, Kato C, Bartlett DH. 2014. Novel psychropiezophilic Oceanospirillales species *Profundimonas piezophila* gen. nov., sp. nov., isolated from the deep-sea environment of the Puerto Rico trench. Appl Environ Microbiol 80:54–60. doi:10.1128/AEM.02288-13 PMC3911031 24123740

[123] Verna C, Ramette A, Wiklund H, Dahlgren TG, Glover AG, Gaill F, Dubilier N. 2010. High symbiont diversity in the bone-eating worm *Osedax mucofloris* from shallow whale-falls in the North Atlantic. Environ Microbiol 12:2355–2370. doi:10.1111/j.1462-2920.2010.02299.x 21966925

[124] Zhang Y, Yu X, Chen Z, Wang Q, Zuo J, Yu S, Guo R. 2023. A review of seagrass bed pollution. Water (Basel) 15:3754. doi:10.3390/w15213754

[125] Yang Y, Sun J, Chen C, Zhou Y, Van Dover CL, Wang C, Qiu J-W, Qian P-Y. 2022. Metagenomic and metatranscriptomic analyses reveal minor-yet-crucial roles of gut microbiome in deep-sea hydrothermal vent snail. Anim Microbiome 4:3. doi:10.1186/s42523-021-00150-z PMC8722025 34980289

[126] Alamoudi R, Barozzi A, Michoud G, Van Goethem MW, Odobel C, Chen Y, Marasco R, Daffonchio D. 2025. Metabolic redundancy and specialisation of novel sulfide-oxidizing Sulfurimonas and Sulfurovum along the brine-seawater interface of the Kebrit Deep. Environ Microbiome 20:19. doi:10.1186/s40793-025-00669-7 PMC11800652 39910644

[127] Cai P, Ning Z, Zhang M. 2025. Carbon sink potential of sulfur-oxidizing bacteria in groundwater at petroleum-contaminated sites. Microorganisms 13:1688. doi:10.3390/microorganisms13071688 PMC12298773 40732196

[128] Boscaro V, Holt CC, Van Steenkiste NWL, Herranz M, Irwin NAT, Àlvarez-Campos P, Grzelak K, Holovachov O, Kerbl A, Mathur V, Okamoto N, Piercey RS, Worsaae K, Leander BS, Keeling PJ. 2022. Microbiomes of microscopic marine invertebrates do not reveal signatures of phylosymbiosis. Nat Microbiol 7:810–819. doi:10.1038/s41564-022-01125-9 35618773

[129] Morgan SG, Fisher JL, McAfee ST, Largier JL, Miller SH, Sheridan MM, Neigel JE. 2014. Transport of crustacean larvae between a low-inflow estuary and coastal waters. Estuaries and Coasts 37:1269–1283. doi:10.1007/s12237-014-9772-y

[130] Sorte CJB, Stachowicz JJ. 2011. Patterns and processes of compositional change in a California epibenthic community. Mar Ecol Prog Ser 435:63–74. doi:10.3354/meps09234

[131] de Jonge VN, Bouwman LA. 1977. A simple density separation technique for quantitative isolation of meiobenthos using the colloidal silica Ludox-TM. Mar Biol 42:143–148. doi:10.1007/BF00391564

[132] Somerfield PJ, Warwick RM. 2013. Meiofauna techniques, p 253–284. In Methods for the study of marine benthos. John Wiley & Sons, Ltd, Oxford, UK.

[133] Fonseca V, Packer M, Carvalho G, Power D, Lambshead J, Creer S. 2011. Isolation of marine meiofauna from sandy sediments: from decanting to DNA extraction. Protoc Exch. doi:10.1038/nprot.2010.157

[134] Pereira TJ, De Santiago A, Schuelke T, Hardy SM, Bik HM. 2020. The impact of intragenomic rRNA variation on metabarcoding‐derived diversity estimates: a case study from marine nematodes. Environ DNA 2:519–534. doi:10.1002/edn3.77

[135] De Santiago A, Pereira TJ, Ferrero TJ, Barnes N, Lallias D, Creer S, Bik HM. 2025. Persistent gaps and errors in reference databases impede ecologically meaningful taxonomy assignments in 18S rRNA studies: a case study of terrestrial and marine nematodes. Environ DNA 7:e70080. doi:10.1002/edn3.70080 PMC12456427 40994536

[136] Caporaso JG, Lauber CL, Walters WA, Berg-Lyons D, Huntley J, Fierer N, Owens SM, Betley J, Fraser L, Bauer M, Gormley N, Gilbert JA, Smith G, Knight R. 2012. Ultra-high-throughput microbial community analysis on the Illumina HiSeq and MiSeq platforms. ISME J 6:1621–1624. doi:10.1038/ismej.2012.8 PMC3400413 22402401

[5] Creer S, Fonseca VG, Porazinska DL, Giblin‐davis RM, Sung W, Power DM, Packer M, Carvalho GR, Blaxter ML, Lambshead PJD, Thomas WK. 2010. Ultrasequencing of the meiofaunal biosphere: practice, pitfalls and promises. Mol Ecol 19:4–20. doi:10.1111/j.1365-294X.2009.04473.x 20331766

[138] Djurhuus A, Pitz K, Sawaya NA, Rojas‐Márquez J, Michaud B, Montes E, Muller‐Karger F, Breitbart M. 2018. Evaluation of marine zooplankton community structure through environmental DNA metabarcoding. Limnol Ocean Methods 16:209–221. doi:10.1002/lom3.10237 PMC5993268 29937700

[139] Cordier T, Angeles IB, Henry N, Lejzerowicz F, Berney C, Morard R, Brandt A, Cambon-Bonavita M-A, Guidi L, Lombard F, Arbizu PM, Massana R, Orejas C, Poulain J, Smith CR, Wincker P, Arnaud-Haond S, Gooday AJ, de Vargas C, Pawlowski J. 2022. Patterns of eukaryotic diversity from the surface to the deep-ocean sediment. Sci Adv 8:eabj9309. doi:10.1126/sciadv.abj9309 PMC8816347 35119936

[140] Zhang GK, Chain FJJ, Abbott CL, Cristescu ME. 2018. Metabarcoding using multiplexed markers increases species detection in complex zooplankton communities. Evol Appl 11:1901–1914. doi:10.1111/eva.12694 PMC6231476 30459837

[141] Martin M. 2011. Cutadapt removes adapter sequences from high-throughput sequencing reads. EMBnet J 17:10. doi:10.14806/ej.17.1.200

[142] Callahan BJ, McMurdie PJ, Rosen MJ, Han AW, Johnson AJA, Holmes SP. 2016. DADA2: high-resolution sample inference from Illumina amplicon data. Nat Methods 13:581–583. doi:10.1038/nmeth.3869 PMC4927377 27214047

[143] Bolyen E, Rideout JR, Dillon MR, Bokulich NA, Abnet C, Al-Ghalith GA, Alexander H, Alm EJ, Arumugam M, Asnicar F, et al.. 2018. QIIME 2: reproducible, interactive, scalable, and extensible microbiome data science. PeerJ Preprints. doi:10.7287/peerj.preprints.27295v1 PMC7015180 31341288

[144] Camacho C, Coulouris G, Avagyan V, Ma N, Papadopoulos J, Bealer K, Madden TL. 2009. BLAST+: architecture and applications. BMC Bioinform 10:421. doi:10.1186/1471-2105-10-421 PMC2803857 20003500

[145] Quast C, Pruesse E, Yilmaz P, Gerken J, Schweer T, Yarza P, Peplies J, Glöckner FO. 2013. The SILVA ribosomal RNA gene database project: improved data processing and web-based tools. Nucleic Acids Res 41:D590–D596. doi:10.1093/nar/gks1219 PMC3531112 23193283

[146] Davis NM, Proctor DM, Holmes SP, Relman DA, Callahan BJ. 2018. Simple statistical identification and removal of contaminant sequences in marker-gene and metagenomics data. Microbiome 6:226. doi:10.1186/s40168-018-0605-2 PMC6298009 30558668

[147] Pruesse E, Peplies J, Glöckner FO. 2012. SINA: accurate high-throughput multiple sequence alignment of ribosomal RNA genes. Bioinformatics 28:1823–1829. doi:10.1093/bioinformatics/bts252 PMC3389763 22556368

[148] Beccati A, Gerken J, Quast C, Yilmaz P, Glöckner FO. 2017. SILVA tree viewer: interactive web browsing of the SILVA phylogenetic guide trees. BMC Bioinform 18. doi:10.1186/s12859-017-1841-3 PMC5622420 28964270

[149] McMurdie P.J, Holmes S. 2013. Phyloseq: an R package for reproducible interactive analysis and graphics of microbiome census data. PLoS One 8:e61217. doi:10.1371/journal.pone.0061217 PMC3632530 23630581

[5] McMurdie PJ, Holmes S. 2014. Waste not, want not: why rarefying microbiome data is inadmissible. Comput Biol 10:e1003531. doi:10.1371/journal.pcbi.1003531 PMC3974642 24699258

[151] Fernandes AD, Macklaim JM, Linn TG, Reid G, Gloor GB. 2013. ANOVA-like differential expression (ALDEx) analysis for mixed population RNA-Seq. PLoS One 8:e67019. doi:10.1371/journal.pone.0067019 PMC3699591 23843979

[152] Benjamini Y, Hochberg Y. 1995. Controlling the false discovery rate: a practical and powerful approach to multiple testing. J R Stat Soc Series B Stat Methodol 57:289–300. doi:10.1111/j.2517-6161.1995.tb02031.x

[153] Douglas GM, Maffei VJ, Zaneveld JR, Yurgel SN, Brown JR, Taylor CM, Huttenhower C, Langille MGI. 2020. PICRUSt2 for prediction of metagenome functions. Nat Biotechnol 38:685–688. doi:10.1038/s41587-020-0548-6 PMC7365738 32483366

[154] Bastian M, Heymann S, Jacomy M. 2009. Gephi: an open source software for exploring and manipulating networks. ICWSM 3:361–362. doi:10.1609/icwsm.v3i1.13937

[155] Fruchterman TMJ, Reingold EM. 1991. Graph drawing by force‐directed placement. Softw Pract Exp 21:1129–1164. doi:10.1002/spe.4380211102

[156] Wickham H. 2009. ggplot2: elegant graphics for data analysis. 2nd ed. Springer Publishing Company, Incorporated.

[157] R Core Team. 2023. R: a language and environment for statistical computing. R Foundation for Statistical Computing, Vienna, Austria.

