# Supplementary material for: Location and habitat specificity structure benthic microbial assemblages in a temperate seagrass ecosystem
Source: mSphere. 2026 Aug 12;11(9):e00431-26. doi: 10.1128/msphere.00431-26 (PMC13621858; doi:10.1128/msphere.00431-26)
Supplement: Supplementary file 1 [file msphere.00431-26-s0001.pdf]

Supplemental Figures

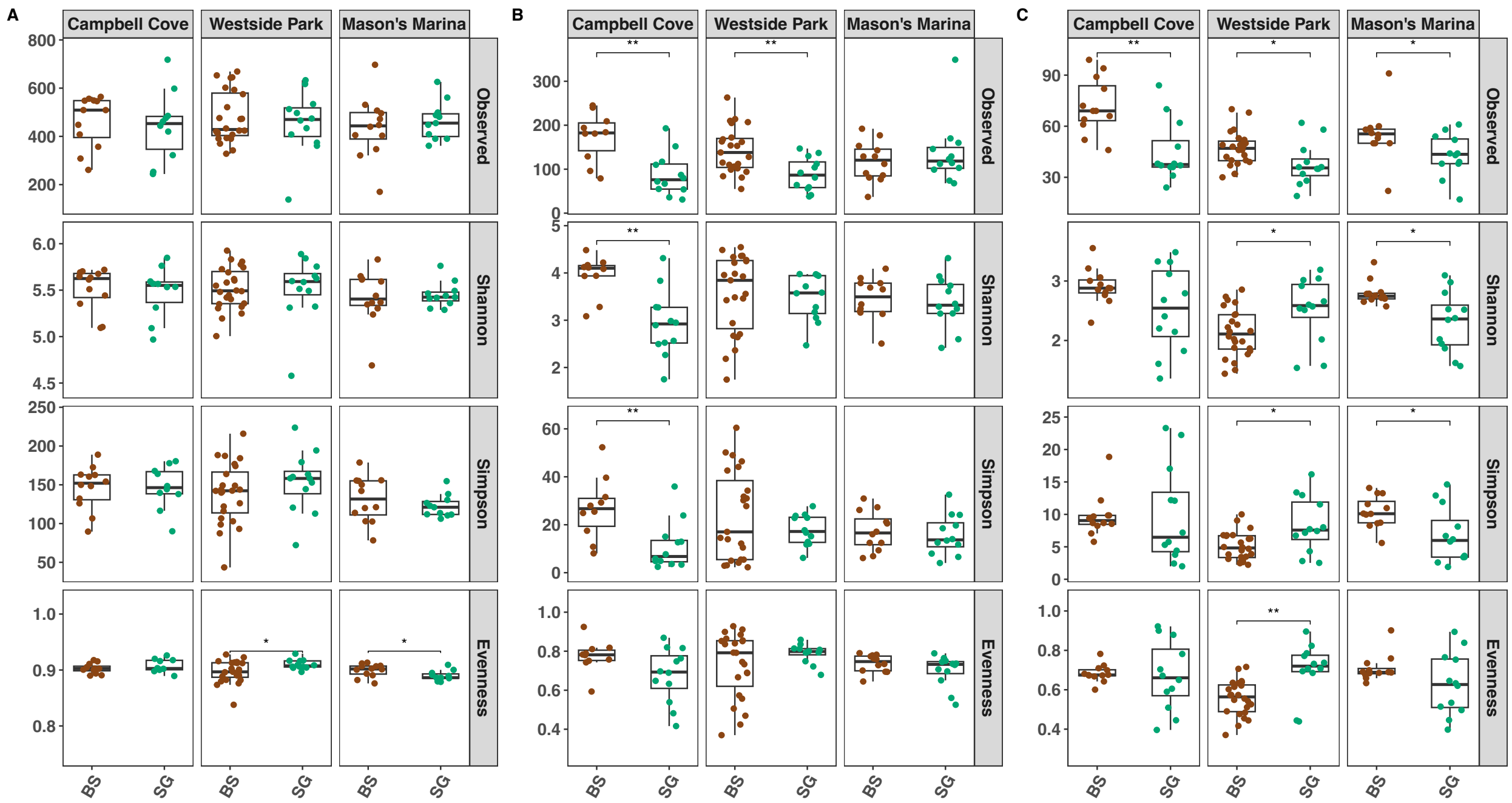

Figure S1: Variation of alpha diversity metrics between bare sediment and seagrass habitats within sites. The Observed (i.e., Richness), Simpson, Shannon, and Evenness diversity metrics were estimated for both habitats sampled at each location for **A**) microbial (16S), **B**) microeukaryotes (18S raw sediment), and **C**) meiofaunal (18S Ludox) communities. Only significant differences ($p < 0.05$) between habitats, based on the Wilcoxon signed-rank test, are shown (Table S3).

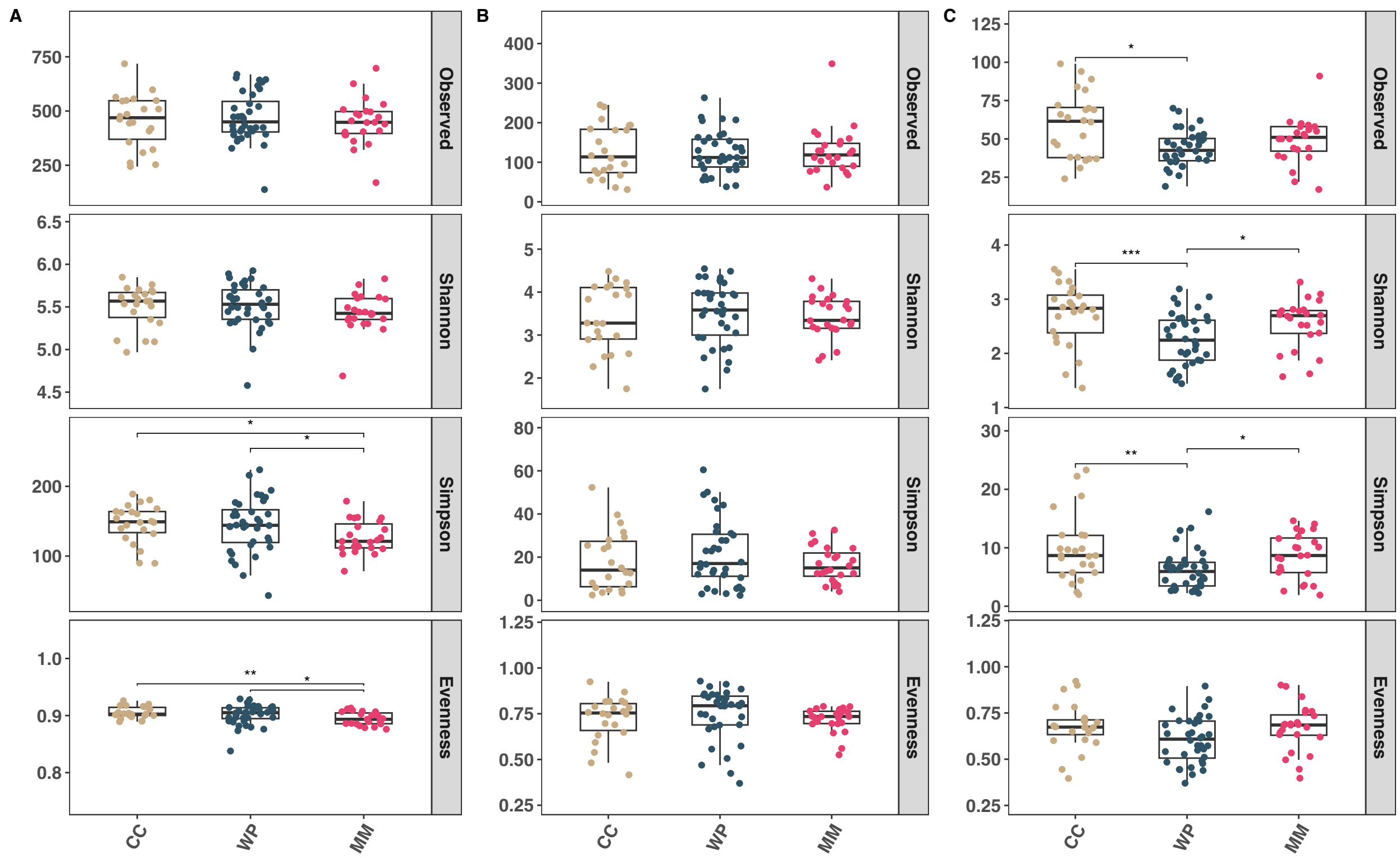

Figure S2: Overall alpha diversity metrics across sites in Bodega Harbor. The Observed (i.e., Richness), Simpson, Shannon, and Evenness diversity metrics were estimated for **A**) microbial (16S), **B**) broad microeukaryote (18S raw sediment), and **C**) meiofaunal (18S Ludox) communities at three sites in Bodega Harbor (CC: Campbell Cove, WP: Westside Park, MM: Mason's Marina). Only significant differences ($p < 0.05$) among sites, identified by pairwise analysis following a Kruskal-Wallis test, are indicated.

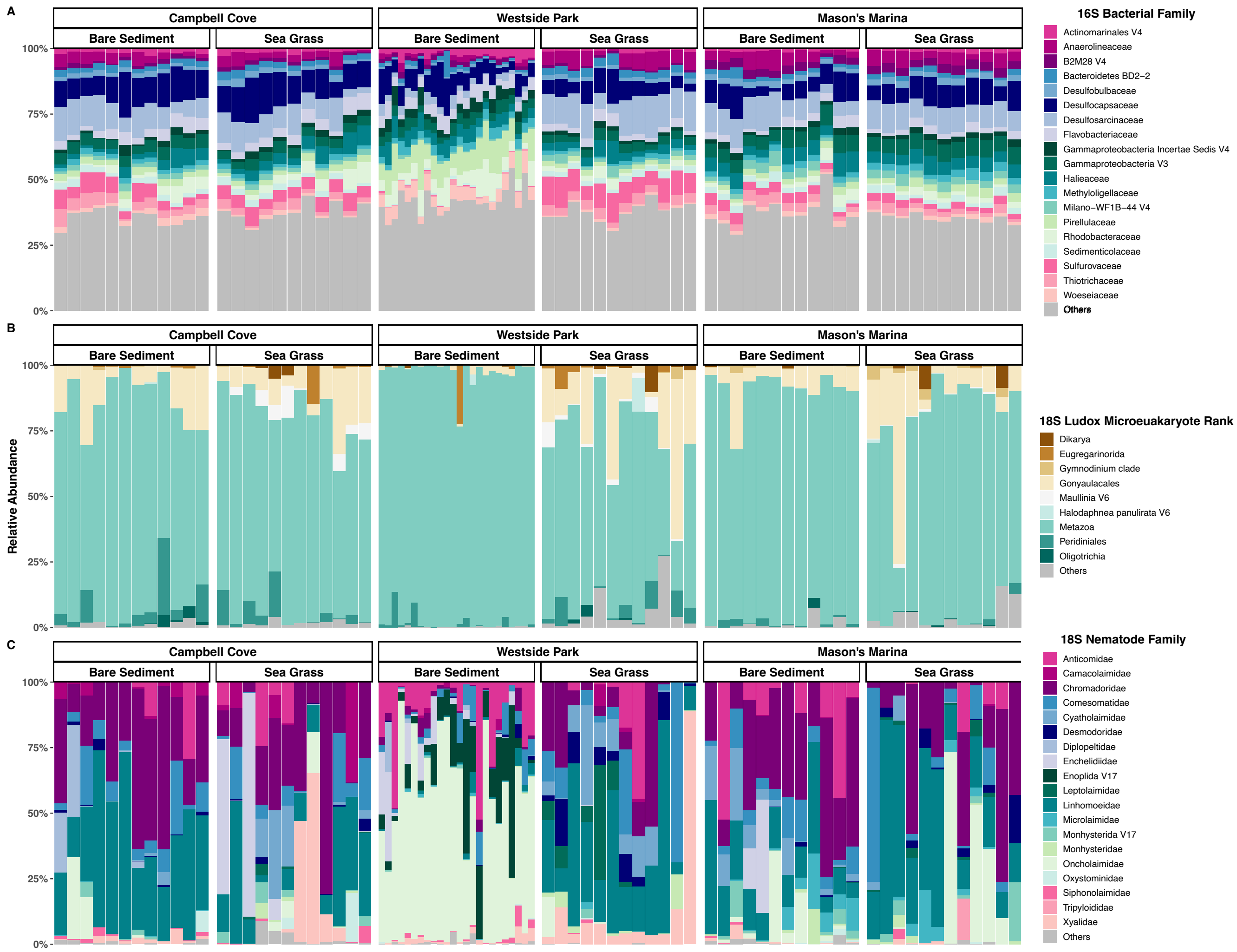

Figure S3: Composition of microbial, microeukaryotic, and nematode communities across sites and habitats at Bodega Harbor. A) Family-level classification for the 19 most abundant bacteria/archaea families (16S). **B**) The 9 most abundant meiofaunal taxa (18S Ludox). **C**) The19 most abundant nematode families identified in meiofaunal samples (18S Ludox). Lower abundance taxa in each dataset were grouped under the category “Others”.

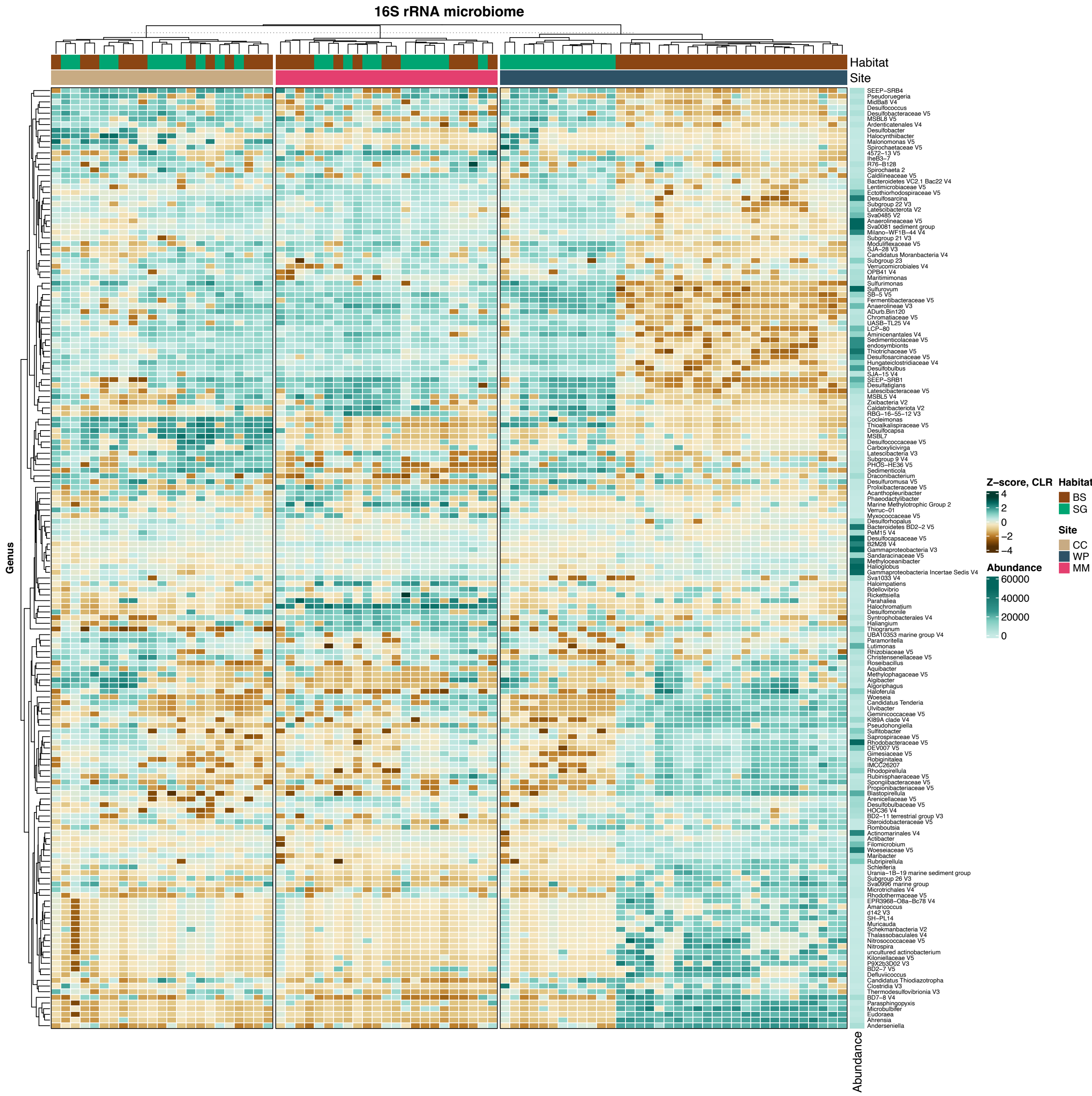

Figure S4. Full results of differential abundance analysis of bacteria and archaea (16S; genus level). Heatmap of differential abundant bacterial/archaeal genera across sample sites (CC: Campbell Cove, WP: Westside Park, MM: Mason's Marina) and habitats (BS: Bare sediment, SG: Seagrass). Analysis was run comparing sample sites. ASV abundances were transformed using the centred-log ratio (CLR). Dark green indicates high abundance; brown indicates low abundance.

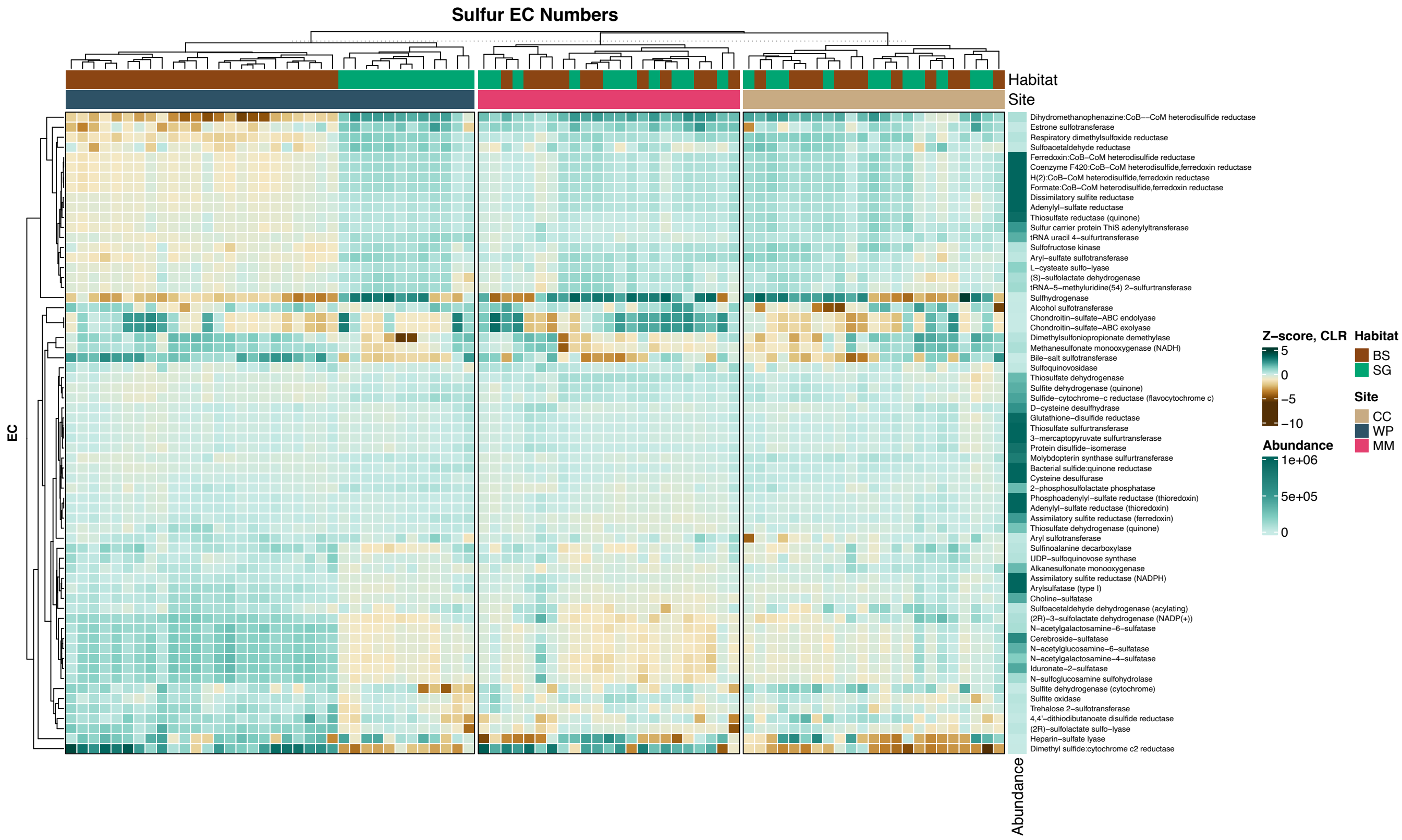

Figure S5. Differential abundance analysis based on predicted functions related to sulfur cycling pathways. The heatmap shows differentially abundant sulfur cycling pathways across sample sites (CC: Campbell Cove, WP: Westside Park, MM: Mason's Marina) and habitats (BS: Bare sediment, SG: Sea grass). Differential abundance analysis comparing sample sites was first run on all predicted functions (i.e., EC numbers), after which only significant (kw.ep < 0.05) sulfur specific pathways were selected. The abundance of predicted functions (EC numbers) was transformed using the centred-log ratio (CLR). Dark green indicates high abundance, while brown indicates low abundance. A complete list of results for all metabolic pathways is provided in Table S5.

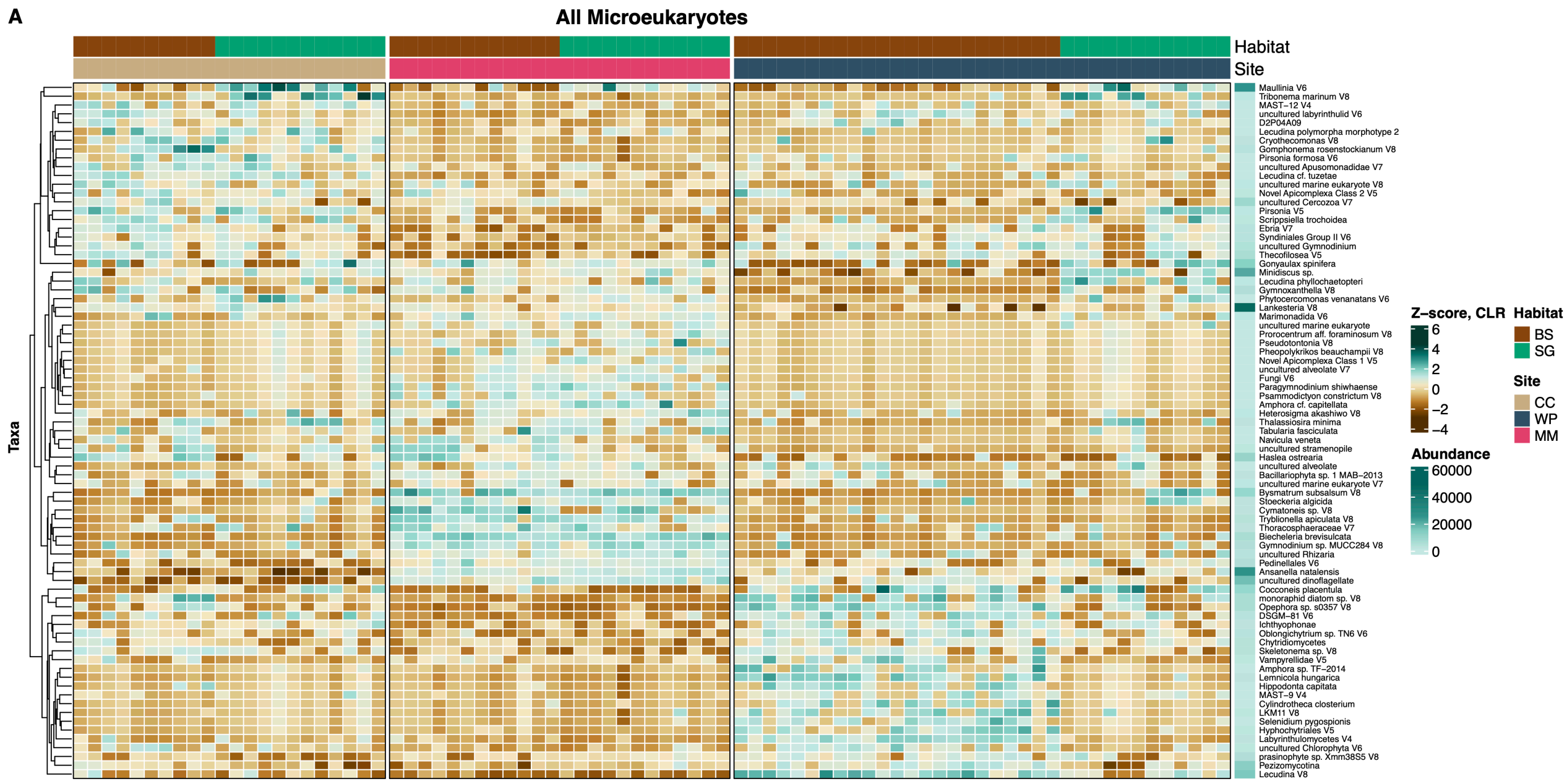

Figure S6. Differential abundance analysis of microeukaryotes (18S raw sediment; V9 level). Heatmap of differentially abundant microeukaryote taxa across sample sites (CC: Campbell Cove, WP: Westside Park, MM: Mason's Marina) and habitats (BS: Bare sediment, SG: Seagrass). Analysis was run comparing sample sites in the ALDEx2 software package. ASV abundances were transformed using the centered-log ratio (CLR). Dark green indicates high abundance; brown indicates low abundance.

Supplemental Tables (Excel File)

All supplemental tables are provided in a separate Microsoft Excel file; Table numbers refer to separate tabs within this file

Table S1. Environmental variables associated with sites and habitats sampled at Bodega Harbor. The variables included sulfate, total organic carbon (TOC), sediment texture (percentages of sand, silt, and clay). GPS coordinates for each site and habitat are provided, as well as disturbance levels (i.e., none-low [NL], low-medium [LM], medium-high [MH]), determined by their distance from anthropogenic activity. Mean values of environmental variables across sites and habitats are included. KW analysis results for comparisons between sites, habitats, and individual habitats within sites are presented (significant values in bold).

Table S2. Sequencing depth for samples representing sites and habitats sampled at Bodega Harbor. The datasets include 16S (bacteria/archaea), 18S raw sediment (broad microeukaryotes), and 18S Ludox (meiofauna). Data presented are the number of reads (i.e., input) fed to and retained (non-chimeric) by DADA2 as well as the number of unique DNA sequences (ASVs). Final counts of reads and ASVs (i.e., after *decontam*) were used in statistical and ecological analyses. Samples highlighted in red had low read count or possible errors and were excluded from the respective datasets. Samples highlighted in blue are controls and blanks.

Table S3. Summary of alpha diversity metrics. Mean, standard deviation (SD), and KW test results (X^2 and p-value) are presented for each alpha diversity index (Observed, Shannon, Simpson, and Evenness) across all datasets (16S, 18S raw sediment, and 18S Ludox) are provided. Metrics were compared among sites, between habitats, and between habitats within sites for each dataset. Significant p-values are highlighted in bold.

Table S4. Summary PERMANOVA results, including pairwise comparisons across datasets (Environmental, 16S, 18S Ludox, and 18S raw sediment). Abbreviations: df: degrees of freedom; SS: sum of squares; R2: coefficient of determination; F: F statistic; Pr(>F): p-value; Res: residual. Sites:Campbell Cove (CC), Westside Park (WP), and Mason's Marina (MM). Habitats: Bare Sediment (BS) and Seagrass (SG).

Table S5. Significant differentially abundant predicted functions (i.e., 16S dataset) among sites (kw.ep < 0.05). Highlighted functions related to sulfur cycling pathways and were presented in Figure S4. Specific sulfur functions of interest were subsetted and further explored for bacterial taxa that are host-associated or hypothesized to be symbionts.

Table S6. Complete node and edge table for significant correlations (p-value < 0.05) in the co-occurrence network analysis. The weight represents the Spearman's correlation coefficient.
